# Supplementary figures and images for: Genetic and functional diversification of chemosensory pathway receptors in mosquito-borne filarial nematodes
Source: PLoS Biol. 2020 Jun 8;18(6):e3000723. doi: 10.1371/journal.pbio.3000723 (PMC7302863; doi:10.1371/journal.pbio.3000723)

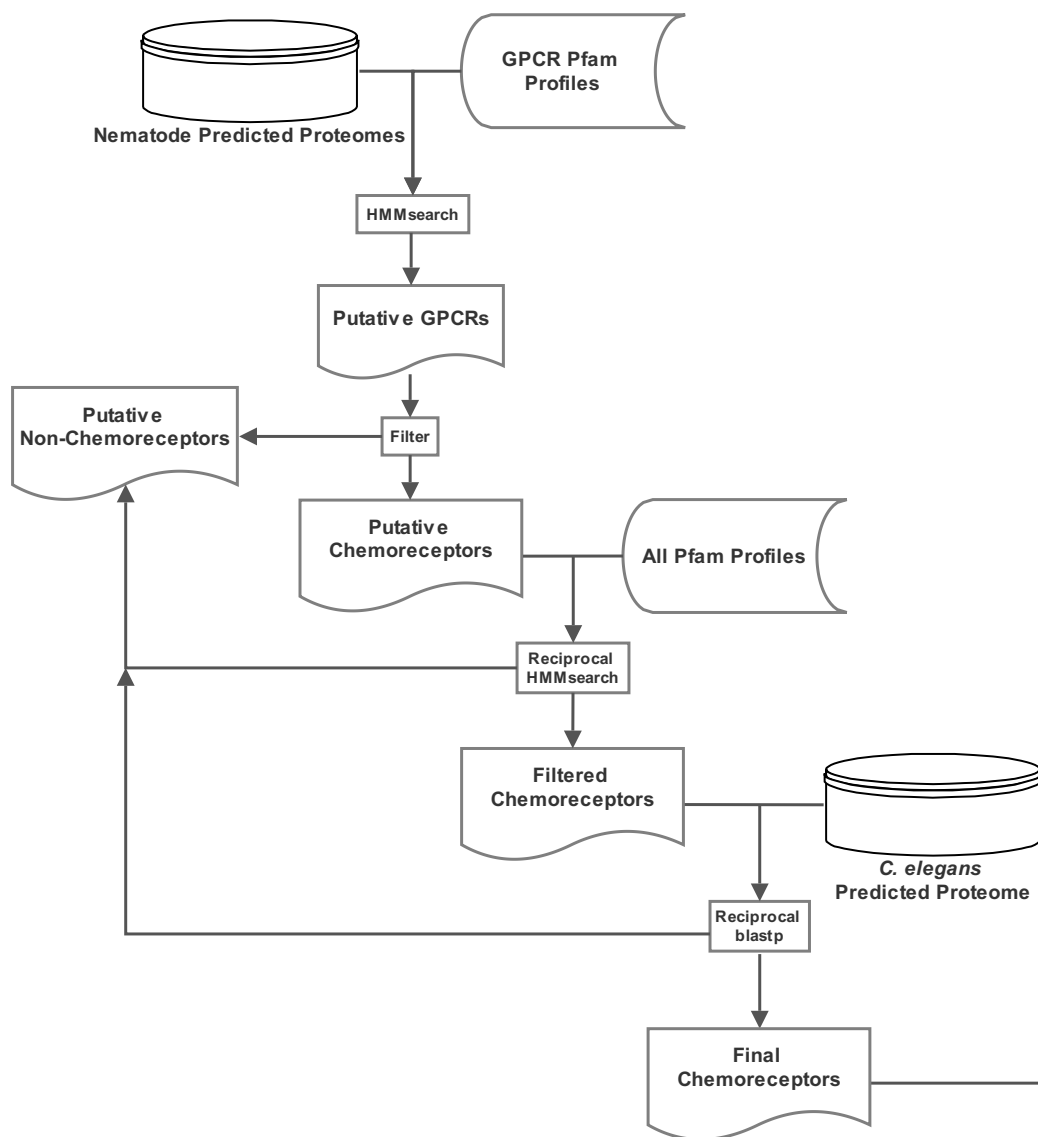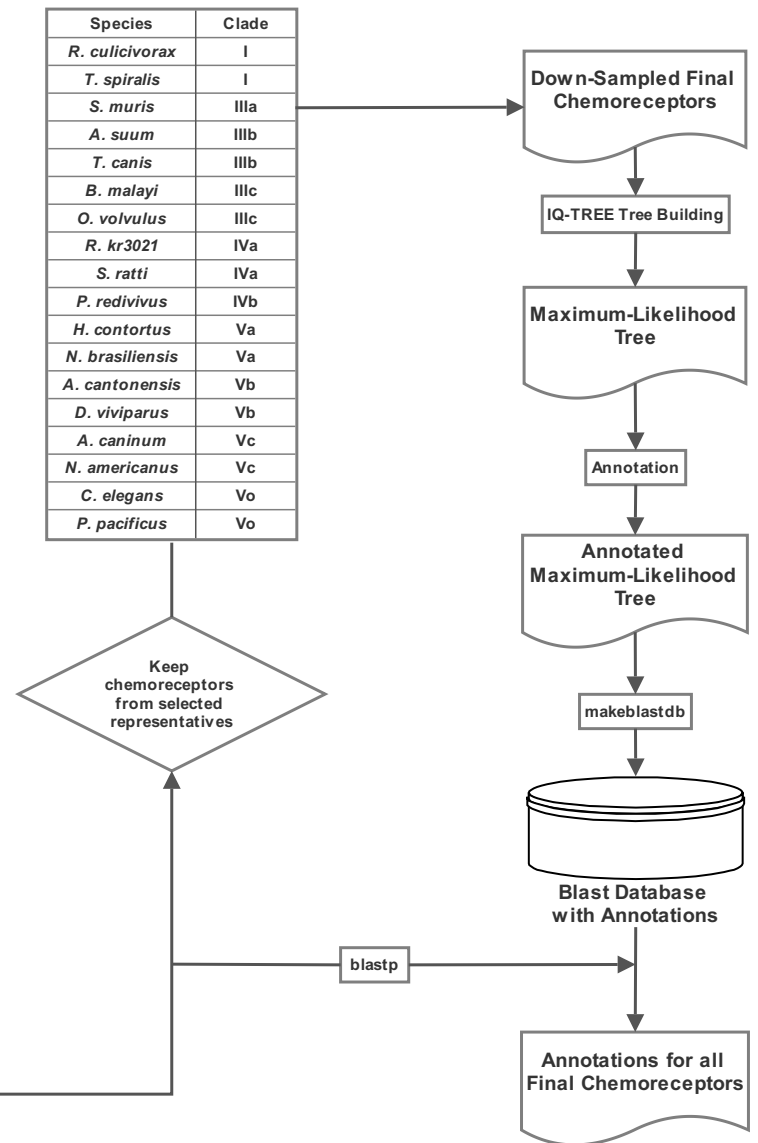

Supplement: S1 Fig — (PDF) [file pbio.3000723.s006.pdf]

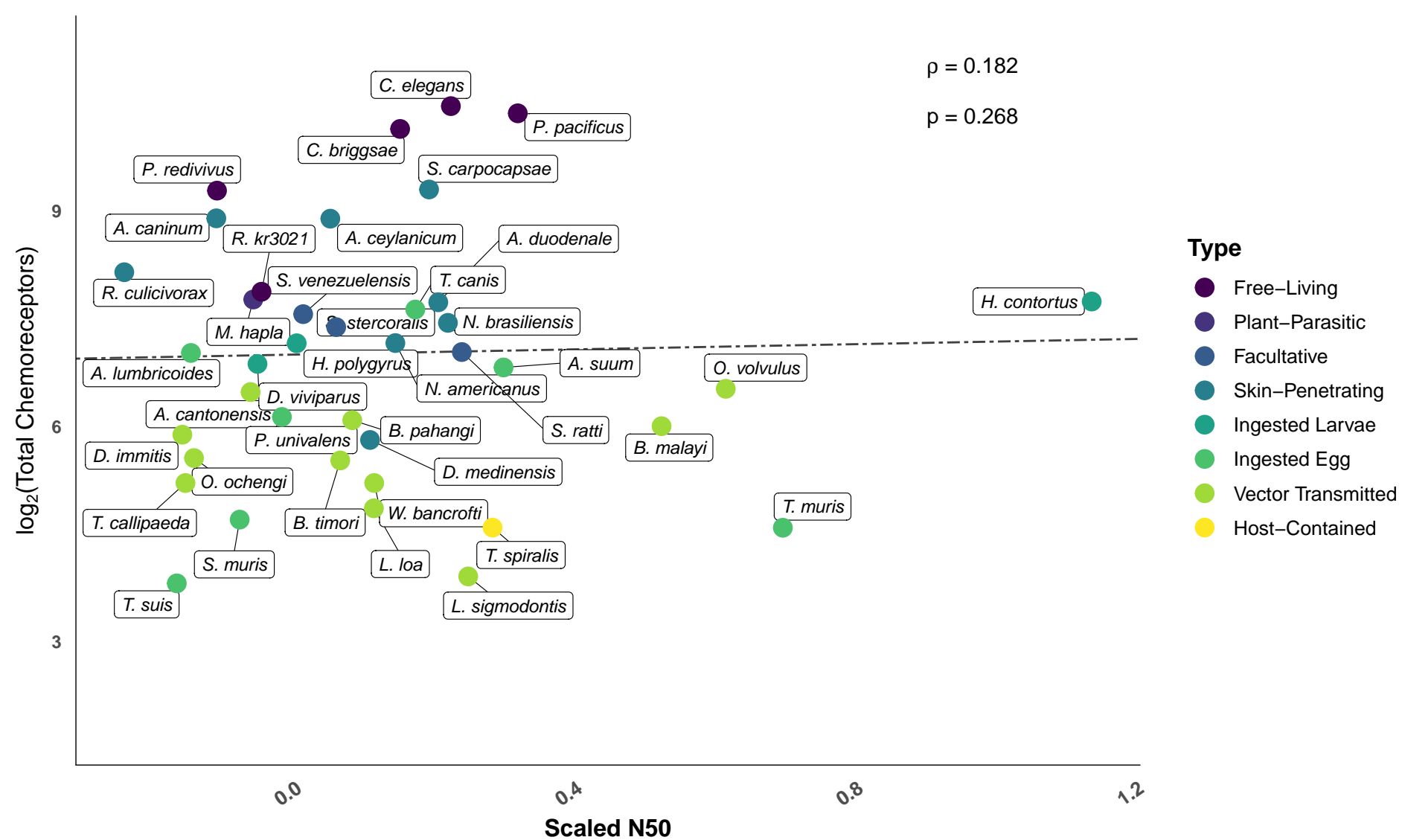

Supplement: S2 Fig — The number of chemoreceptors in a given genome is not correlated to genome contiguity as measured by N50 (Spearman’s rank-order correlation, ρ = 0.182, p = 0.268). Raw data can be found at https://github.com/zamanianlab/BrugiaChemo-ms. (PDF) [file pbio.3000723.s007.pdf]

A

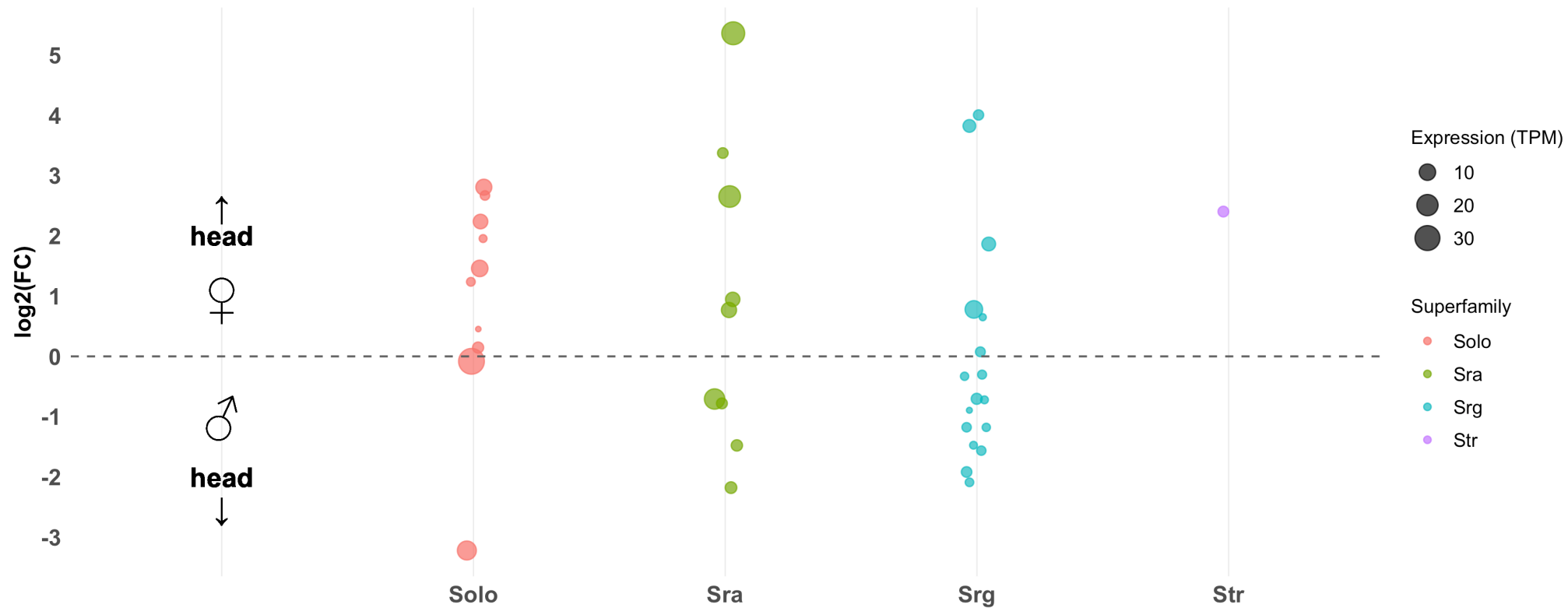

B

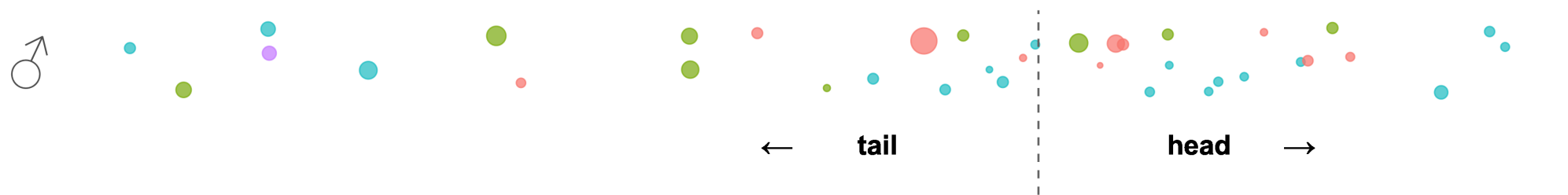

Supplement: S3 Fig — Chemoreceptors are colored by superfamily annotation. Raw data can be found at https://github.com/zamanianlab/BrugiaChemo-ms. RNA-seq, RNA sequencing. (PDF) [file pbio.3000723.s008.pdf]

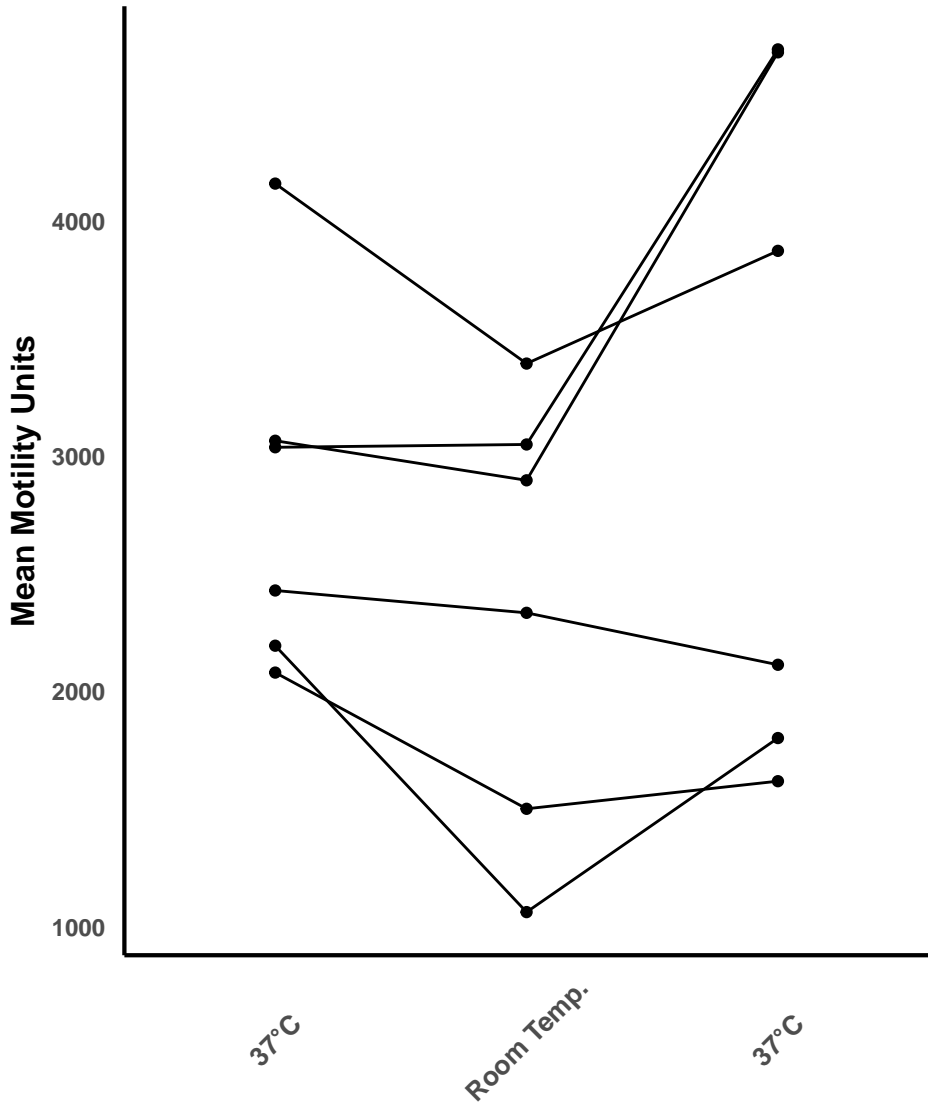

Supplement: S4 Fig — Worms move less after being cooled to room temperature, and motility subsequently increases after returning to 37°C. Raw data can be found at https://github.com/zamanianlab/BrugiaChemo-ms. L3, third stage larvae. (PDF) [file pbio.3000723.s009.pdf]

**A**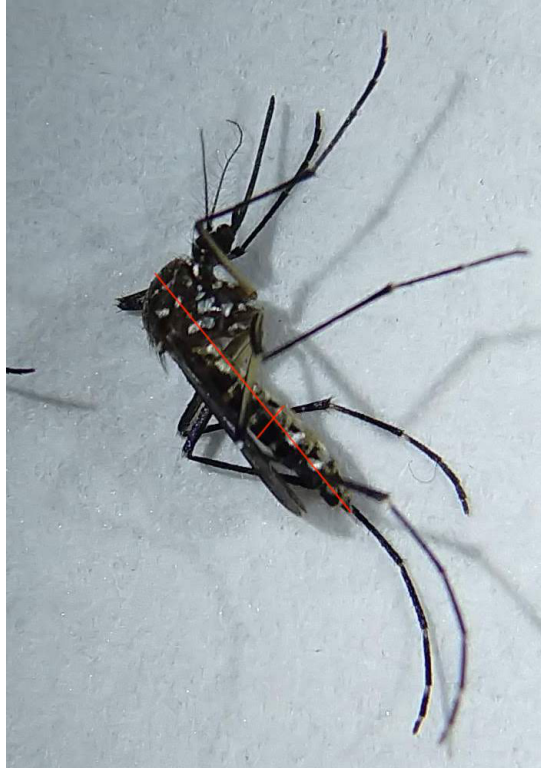**B**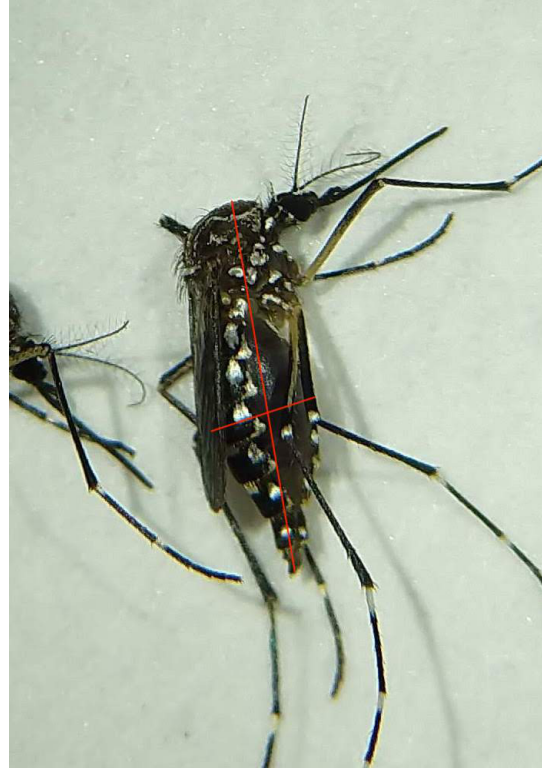**C**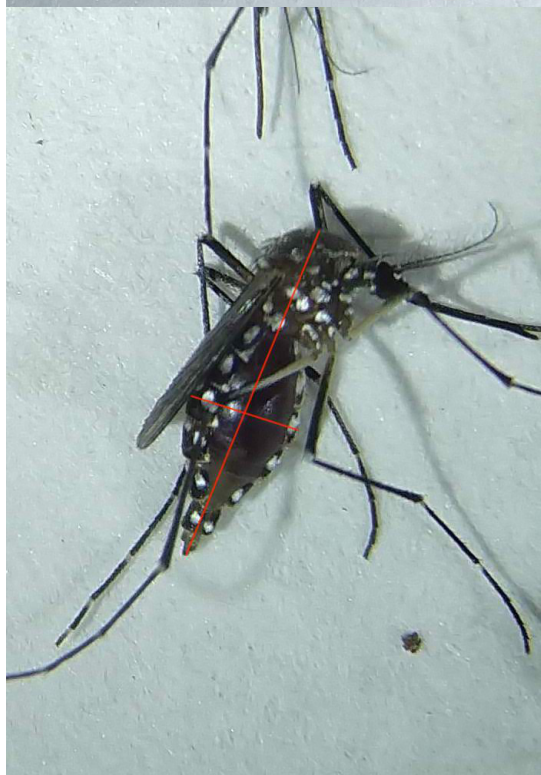**D**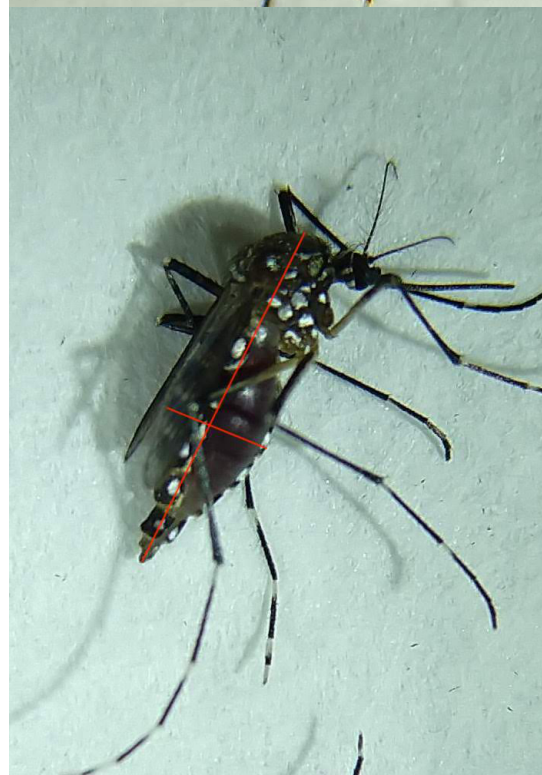

Supplement: S5 Fig — (A) Unfed. (B) Fed with unsupplemented blood. (C) Fed with blood supplemented with 5 mM NAM. (D) Fed with blood supplemented with 25 mM NAM. NAM, nicotinamide. (PDF) [file pbio.3000723.s010.pdf]

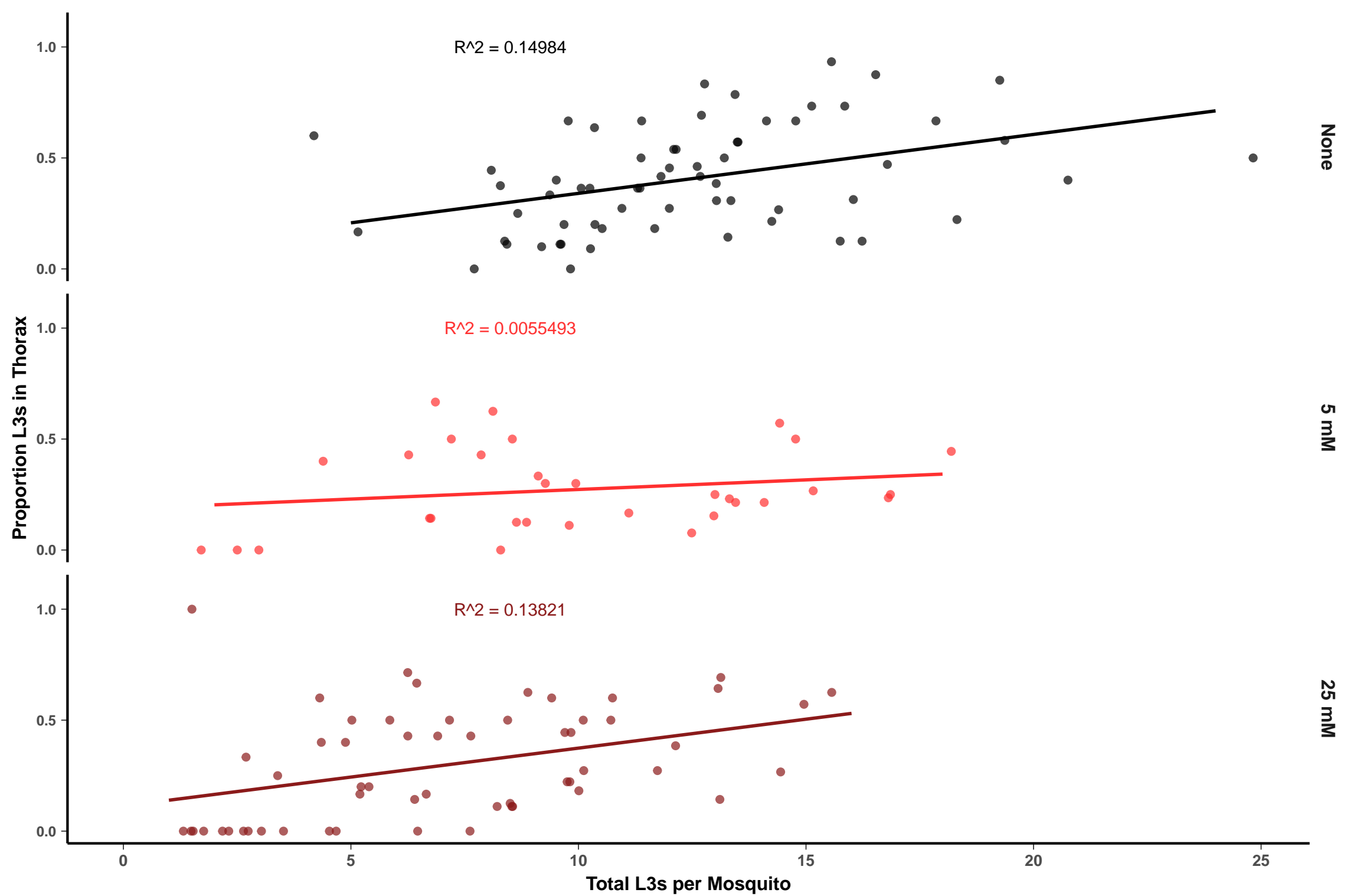

Supplement: S6 Fig — The proportion of L3s recovered in the mosquito thorax does not correlate with the total L3s recovered per mosquito. Raw data can be found at https://github.com/zamanianlab/BrugiaChemo-ms. L3, third stage larvae. (PDF) [file pbio.3000723.s011.pdf]

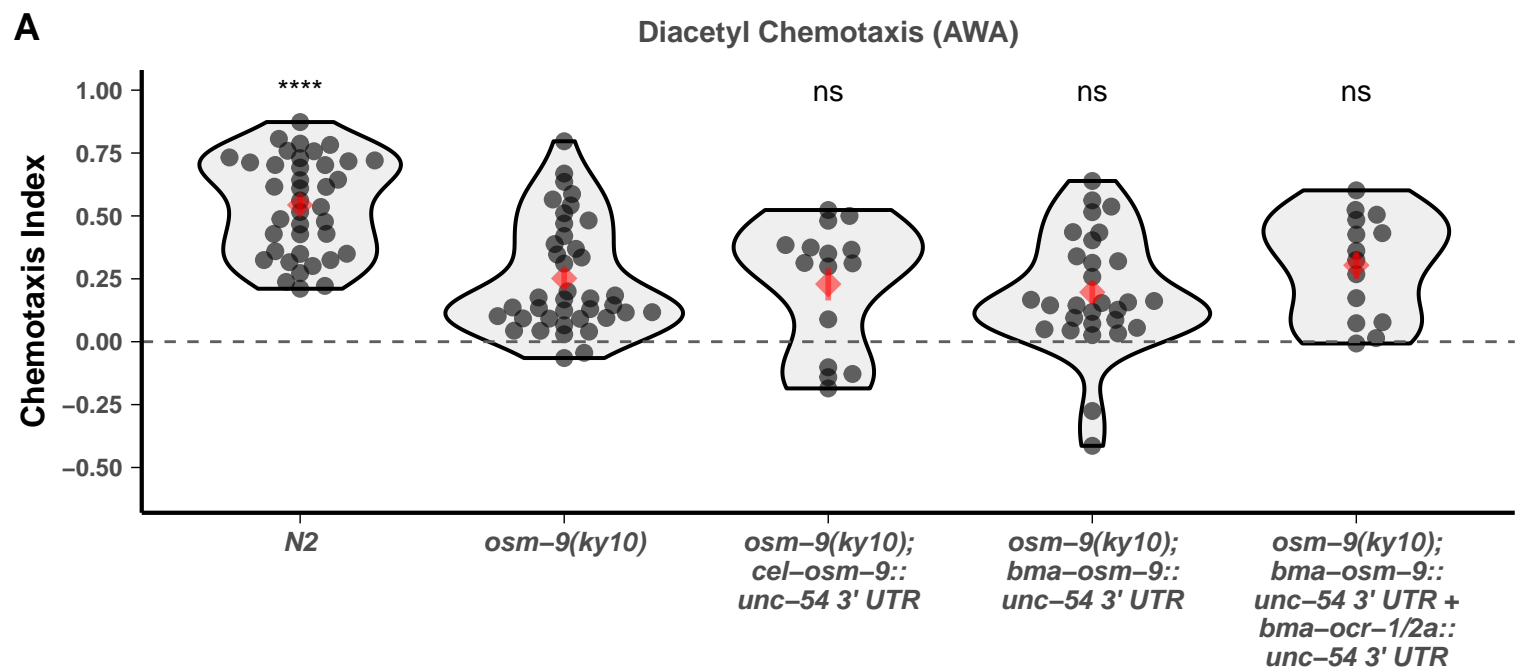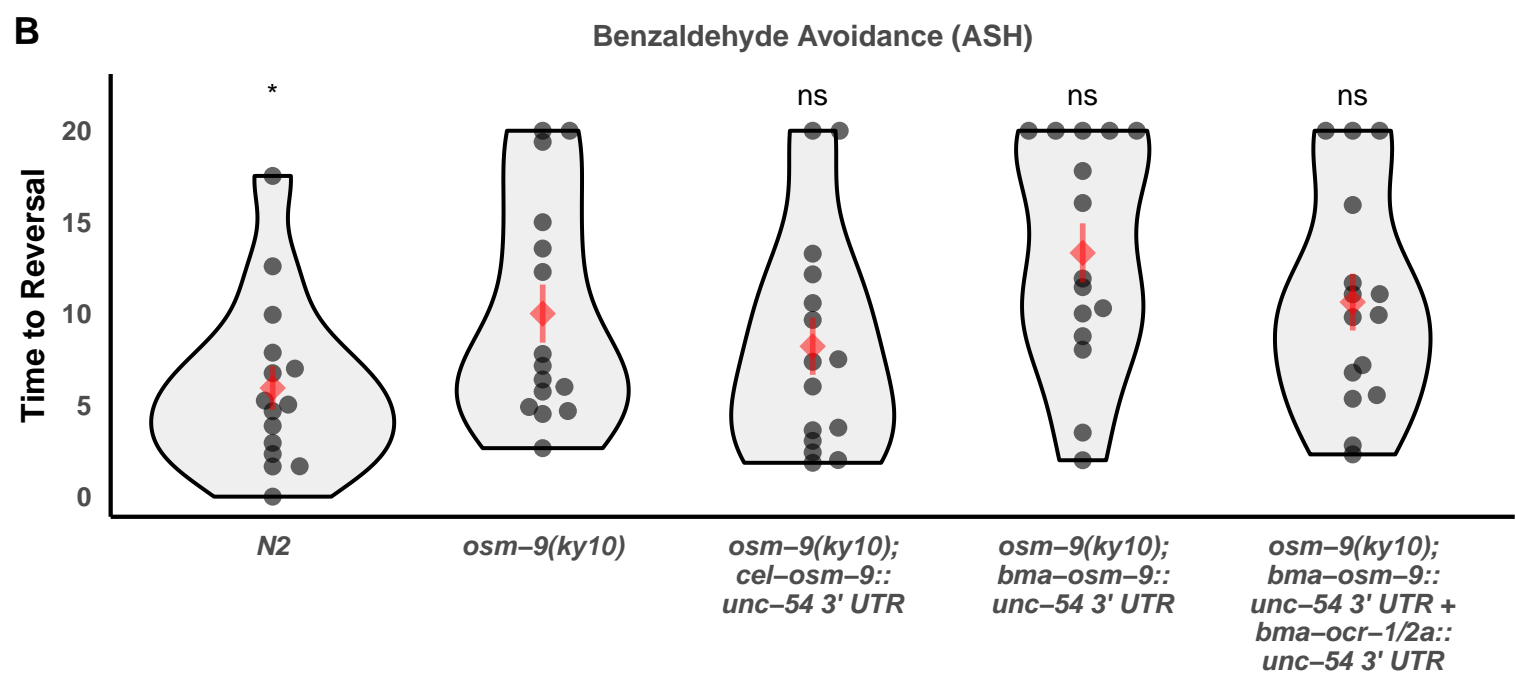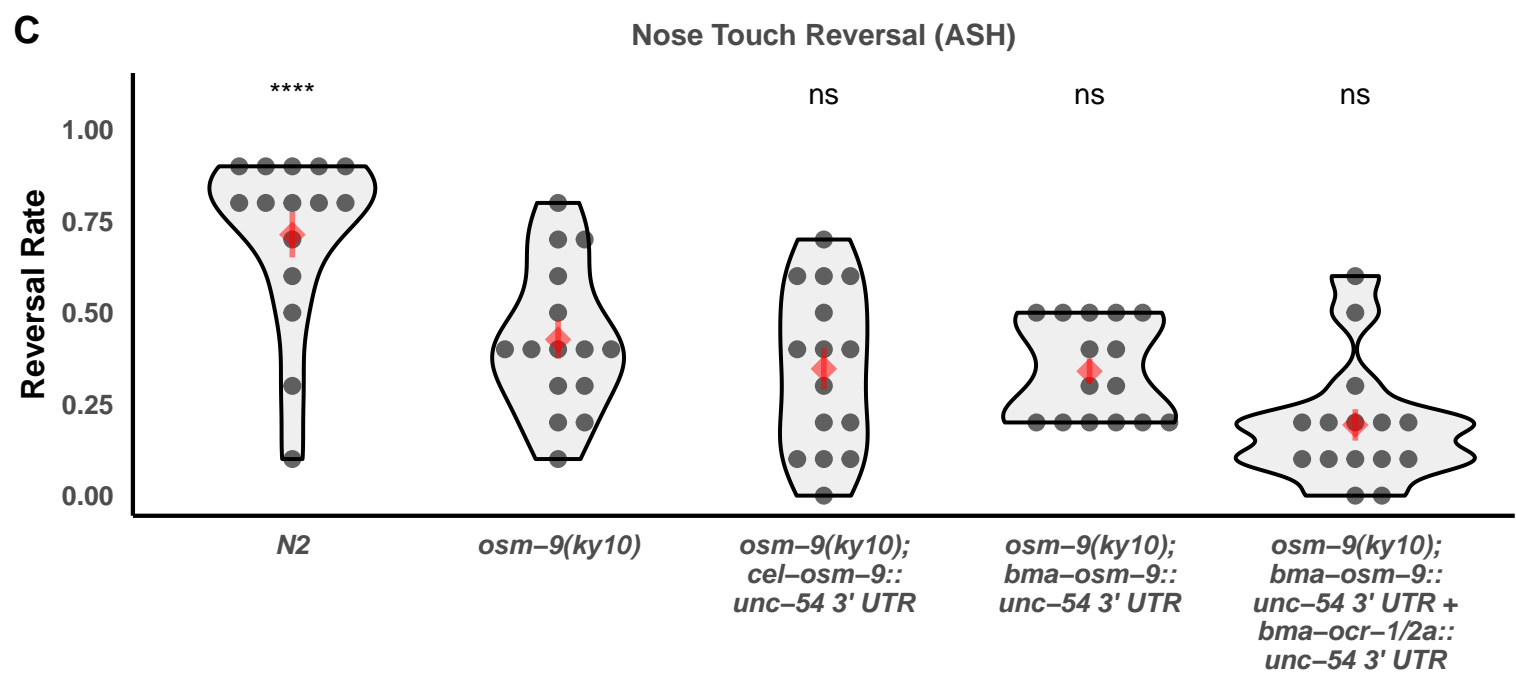

Supplement: S11 Fig — Strains with the unc-54 3′ UTR were unable to rescue (A) defects in chemotaxis to diacetyl, (B) avoidance of concentrated benzaldehyde, or (C) reversal after light nose touch. Raw data can be found at https://github.com/zamanianlab/BrugiaChemo-ms. (PDF) [file pbio.3000723.s016.pdf]

# Diacetyl Chemotaxis (AWA)

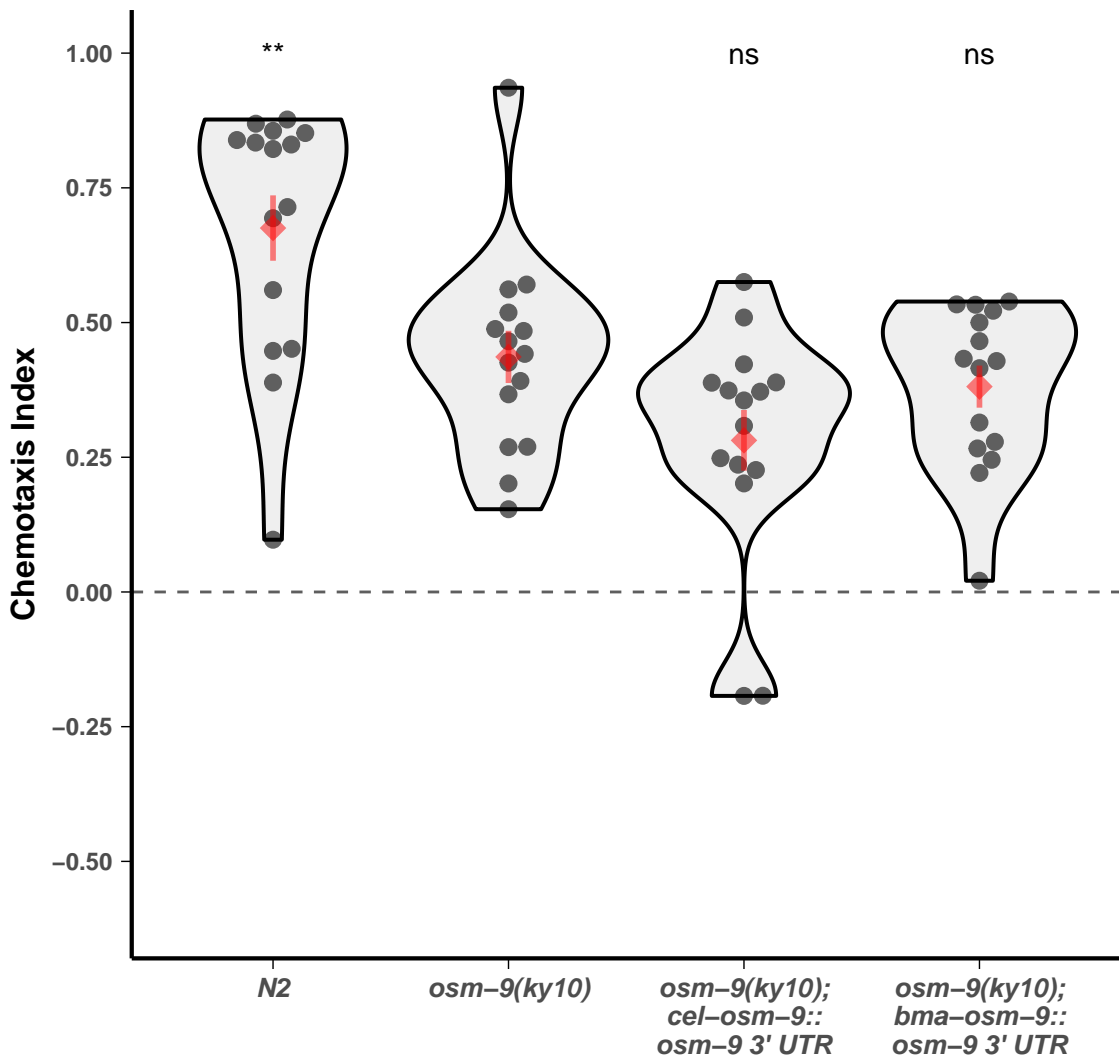

Supplement: S12 Fig — Strains with the osm-9 3′ UTR were unable to rescue defects in chemotaxis to diacetyl. Raw data can be found at https://github.com/zamanianlab/BrugiaChemo-ms. (PDF) [file pbio.3000723.s017.pdf]
