# Supplementary material for: Genetic and functional diversification of chemosensory pathway receptors in mosquito-borne filarial nematodes
Source: PLoS Biol. 2020 Jun 8;18(6):e3000723. doi: 10.1371/journal.pbio.3000723 (PMC7302863; doi:10.1371/journal.pbio.3000723)
Supplement: S7 Fig — The missing splice acceptor in the predicted gene model (Bm-osm-9_Bm1711.1) can be seen on the line starting with nucleotide 2,401. (PDF) [file pbio.3000723.s012.pdf]

```
                                 1
Bm-osm-9_Bm1711.1                ATGGGACAAC TAAAGAGCAA AATTTTGCAC CAAACTGGAG GAGAAGATAT TGACAATTTA AAAGAAGATC CTGACGATCA ATGGTCGAAT TTATATCGTG AACGTAAAAA GAATCACCTT
Bm-osm-9_clone                   ATGGGACAAC TAAAGAGCAA AATTTTGCAC CAAACTGGAG GAGAAGATAT TGACAATTTA AAAGAAGATC CTGACGATCA ATGGTCGAAT TTATATCGTG AACGTAAAAA GAATCACCTT
i3_LQ_NR42497|c19820/f1p1/3020   ATGGGACAAC TAAAGAGCAA AATTTTGCAC CAAACTGGAG GAGAAGATAT TGACAATTTA AAAGAAGATC CTGACGATCA ATGGTCGAAT TTATATCGTG AACGTAAAAA GAATCACCTT
i2_LQ_NR42497|c51951/f1p6/3009   ATGGGACAAC TAAAGAGCAA AATTTTGCAC CAAACTGGAG GAGAAGATAT TGACAATTTA AAAGAAGATC CTGACGATCA ATGGTCGAAT TTATATCGTG AACGTAAAAA GAATCACCTT

                               121
Bm-osm-9_Bm1711.1                TACAAATGGG TTGGCATGCG CAGTGGTGGT GAACTGTTGG CTGCTTTTGA AAAGGAAGGT GAAGATGGAG TTTTAAAGTT TGCGAACGAA AAGCTTATTT CAATGATGTA TGATGATGGT
Bm-osm-9_clone                   TACAAATGGG TTGGCATGCG CAGTGGTGGT GAACTGTTGG CTGCTTTTGA AAAGGAAGGT GAAGATGGAG TTTTAAAGTT TGCGAACGAA AAGCTTATTT CAATGATGTA TGATGATGGT
i3_LQ_NR42497|c19820/f1p1/3020   TACAAATGGG TTGGCATGCG CAGTGGTGGT GAACTGTTGG CTGCTTTTGA AAAGGAAGGT GAAGATGGAG TTTTAAAGTT TGCGAACGAA AAGCTTATTT CAATGATGTA TGATGATGGT
i2_LQ_NR42497|c51951/f1p6/3009   TACAAATGGG TTGGCATGCG CAGTGGTGGT GAACTGTTGG CTGCTTTTGA AAAGGAAGGT GAAGATGGAG TTTTAAAGTT TGCGAACGAA AAGCTTATTT CAATGATGTA TGATGATGGT

                               241
Bm-osm-9_Bm1711.1                GCATCACCGC AAATGATTCG TTTTACAGAC TATGCAAAAT GGAAAAAGAC AACTAATGTA CAGCTGGGTA AAACAGAATC AAACTCTGTT GGCCAATTTG GTAGCAAATT CCGTGAACAT
Bm-osm-9_clone                   GCATCACCGC AAATGATTCG TTTTACAGAC TATGCAAAAT GGAAAAAGAC AACTAATGTA CAGCTGGGTA AAACAGAATC AAACTCTGTT GGCCAATTTG GTAGCAAATT CCGTGAACAT
i3_LQ_NR42497|c19820/f1p1/3020   GCATCACCGC AAATGATTCG TTTTACAGAC TATGCAAAAT GGAAAAAGAC AACTAATGTA CAGCTGGGTA AAACAGAATC AAACTCTGTT GGCCAATTTG GTAGCAAATT CCGTGAACAT
i2_LQ_NR42497|c51951/f1p6/3009   GCATCACCGC AAATGATTCG TTTTACAGAC TATGCAAAAT GGAAAAAGAC AACTAATGTA CAGCTGGGTA AAACAGAATC AAACTCTGTT GGCCAATTTG GTAGCAAATT CCGTGAACAT

                               361
Bm-osm-9_Bm1711.1                CTTGGTCAGT GGAGGTTGAA CAAACGTGGT GTGGAAGGTG AGACGATAAT ACACCTATTG CTGAATCGTG AGGAACCAAT GTGTAGTGAA ATTGCTAGAA TTTTAATAAC ACGTTATCCA
Bm-osm-9_clone                   CTTGGTCAGT GGAGGTTGAA CAAACGTGGT GTGGAAGGTG AGACGATAAT ACACCTATTG CTGAATCGTG AGGAACCAAT GTGTAGTGAA ATTGCTAGAA TTTTAATAAC ACGTTATCCA
i3_LQ_NR42497|c19820/f1p1/3020   CTTGGTCAGT GGAGGTTGAA CAAACGTGGT GTGGAAGGTG AGACGATAAT ACACCTATTG CTGAATCGTG AGGAACCAAT GTGTAGTGAA ATTGCTAGAA TTTTAATAAC ACGTTATCCA
i2_LQ_NR42497|c51951/f1p6/3009   CTTGGTCAGT GGAGGTTGAA CAAACGTGGT GTGGAAGGTG AGACGATAAT ACACCTATTG CTGAATCGTG AGGAACCAAT GTGTAGTGAA ATTGCTAGAA TTTTAATAAC ACGTTATCCA

                               481
Bm-osm-9_Bm1711.1                GGCCTTGCTA ATGATATTTA CCTCGGCGAC GAAATGTTCG GTCAAAGTGC ATTACATTTA GCAATTGTTC ATGATGATTA TGAAACTGTT CATTTATTAC TGCAAAACTC AGCAGAAGTG
Bm-osm-9_clone                   GGCCTTGCTA ATGATATTTA CCTCGGCGAC GAAATGTTCG GTCAAAGTGC ATTACATTTA GCAATTGTTC ATGATGATTA TGAAACTGTT CATTTATTAC TGCAAAACTC AGCAGAAGTG
i3_LQ_NR42497|c19820/f1p1/3020   GGCCTTGCTA ATGATATTTA CCTCGGCGAC GAAATGTTCG GTCAAAGTGC ATTACATTTA GCAATTGTTC ATGATGATTA TGAAACTGTT CATTTATTAC TGCAAAACTC AGCAGAAGTG
i2_LQ_NR42497|c51951/f1p6/3009   GGCCTTGCTA ATGATATTTA CCTCGGCGAC GAAATGTTCG GTCAAAGTGC ATTACATTTA GCAATTGTTC ATGATGATTA TGAAACTGTT CATTTATTAC TGCAAAACTC AGCAGAAGTG

                               601
Bm-osm-9_Bm1711.1                AATGCCCGGG CTTGCGGGAC ATTTTTTCTA CCTGAGAATC AAAAGACATC TCGCAAATCA ACTGATTACC AAGGATATGC CTATTATGGC GAATATCCGT TAGCATTTGC TGCTTGTTTT
Bm-osm-9_clone                   AATGCCCGGG CTTGCGGGAC ATTTTTTCTA CCTGAGAATC AAAAGACATC TCGCAAATCA ACTGATTACC AAGGATATGC CTATTATGGC GAATATCCGT TAGCATTTGC TGCTTGTTTT
i3_LQ_NR42497|c19820/f1p1/3020   AATGCCCGGG CTTGCGGGAC ATTTTTTCTA CCTGAGAATC AAAAGACATC TCGCAAATCA ACTGATTACC AAGGATATGC CTATTATGGC GAATATCCGT TAGCATTTGC TGCTTGTTTT
i2_LQ_NR42497|c51951/f1p6/3009   AATGCCCGGG CTTGCGGGAC A-TTTTTCTA CCTGAGAATC AAAAGACATC TCGCAAATCA ACTGATTACC AAGGATATGC CTATTATGGC GAATATCCGT TAGCATTTGC TGCTTGTTTT

                               721
Bm-osm-9_Bm1711.1                GGCAATAAAG ATATCTATGA TTTACTGATA CAATATGGCG CTGATCCAAA TCTACAAGAT ATGTTCGGCA ACACCATTCT ACATATGTGT GTCATCAACT ATAGCAATTC AATGTACAGT
Bm-osm-9_clone                   GGCAATAAAG ATATCTATGA TTTACTGATA CAATATGGCG CTGATCCAAA TCTACAAGAT ATGTTCGGCA ACACCATTCT ACATATGTGT GTCATCAACT ATAGCAATTC AATGTACAGT
i3_LQ_NR42497|c19820/f1p1/3020   GGCAATAAAG ATATCTATGA TTTACTGATA CAATATGGCG CTGATCCAAA TCTACAAGAT ATGTTCGGCA ACACCATTCT ACATATGTGT GTCATCAACT ATAGCAATTC AATGTACAGT
i2_LQ_NR42497|c51951/f1p6/3009   GGCAATAAAG ATATCTATGA TTTACTGATA CAATATGGCG CTGATCCAAA TCTACAAGAT ATGTTCGGCA ACACCATTCT ACATATGTGT GTCATCAACT ATAGCAATTC AATGTACAGT

                               841
Bm-osm-9_Bm1711.1                TATGCAGTAA GGCATTGGGC GAAACCCGCT GATCCAAACA TTGTAAATGC AGCTGGTCTG ACACCATTGA CGCTTGCAAC AAAACTTGGA CGAAAGGATA TCTTTGAGGA AATGCTGGAG
Bm-osm-9_clone                   TATGCAGTAA GGCATTGGGC GAAACCCGCT GATCCAAACA TTGTAAATGC AGCTGGTCTG ACACCATTGA CGCTTGCAAC AAAACTTGGA CGAAAGGATA TCTTTGAGGA AATGCTGGAG
i3_LQ_NR42497|c19820/f1p1/3020   TATGCAGTAA GGCATTGGGC GAAACCCGCT GATCCAAACA TTGTAAATGC AGCTGGTCTG ACACCATTGA CGCTTGCAAC AAAACTTGGA CGAAAGGATA TCTTTGAGGA AATGCTGGAG
i2_LQ_NR42497|c51951/f1p6/3009   TATGCAGTAA GGCATTGGGC GAAACCCGCT GATCCAAACA TTGTAAATGC AGCTGGTCTG ACACCATTGA CGCTTGCAAC AAAACTTGGA CGAAAGGATA TCTTTGAGGA AATGCTGGAG

                               961
Bm-osm-9_Bm1711.1                TTGATGAAAG TCGAGTTTTG GAGATTCAGT GATATGACGT GTAGTGCATA TCCTCTTACA GCTTTGGATA CTATTCGCCC CGATGGCTCA ACAAATTATG ATTCAGCGTT GATGACTGTG
Bm-osm-9_clone                   TTGATGAAAG TCGAGTTTTG GAGATTCAGT GATATGACGT GTAGTGCATA TCCTCTTACA GCTTTGGATA CTATTCGCCC CGATGGCTCA ACAAATTATG ATTCAGCGTT GATGACTGTG
i3_LQ_NR42497|c19820/f1p1/3020   TTGATGAAAG TCGAGTTTTG GAGATTCAGT GATATGACGT GTAGTGCATA TCCTCTTACA GCTTTGGATA CTATTCGCCC CGATGGCTCA ACAAATTATG ATTCAGCGTT GATGACTGTG
i2_LQ_NR42497|c51951/f1p6/3009   TTGATGAAAG TCGAGTTTTG GAGATTCAGT GATATGACGT GTAGTGCATA TCCTCTTACA GCTTTGGATA CTATTCGCCC CGATGGCTCA ACAAATTATG ATTCAGCGTT GATGACTGTG

                              1081
Bm-osm-9_Bm1711.1                ATAAATGGTA GTACCAGTGA ACATTTAGAT ATGATTGGTA GCGAAGTGAT CCAACGACTT CTTGCTGACA AATGGAAAGC TTTTGCATCA AGAAAATTAT TTGAAAGGCT TGGATTGCTC
Bm-osm-9_clone                   ATAAATGGTA GTACCAGTGA ACATTTAGAT ATGATTGGTA GCGAAGTGAT CCAACGACTT CTTGCTGACA AATGGAAAGC TTTTGCATCA AGAAAATTAT TTGAAAGGCT TGGATTGCTC
i3_LQ_NR42497|c19820/f1p1/3020   ATAAATGGTA GTACCAGTGA ACATTTAGAT ATGATTGGTA GCGAAGTGAT CCAACGACTT CTTGCTGACA AATGGAAAGC TTTTGCATCA AGAAAATTAT TTGAAAGGCT TGGATTGCTC
i2_LQ_NR42497|c51951/f1p6/3009   ATAAATGGTA GTACCAGTGA ACATTTAGAT ATGATTGGTA GCGAAGTGAT CCAACGACTT CTTGCTGACA AATGGAAAGC TTTTGCATCA AGAAAATTAT TTGAAAGGCT TGGATTGCTC

                              1201
Bm-osm-9_Bm1711.1                ATTTTACATC TCATATTTTT ATGTTTTGTT GTTTATATGC GGCCGTCTGA ACCGGAGCGA CTTACGTATA AATTAATTGC AACAGAATGG AATGATTGGG TGCGGTTATG CTTTGAAATT
Bm-osm-9_clone                   ATTTTACATC TCATATTTTT ATGTTTTGTT GTTTATATGC GGCCGTCTGA ACCGGAGCGA CTTACGTATA AATTAATTGC AACAGAATGG AATGATTGGG TGCGGTTATG CTTTGAAATT
i3_LQ_NR42497|c19820/f1p1/3020   ATTTTACATC TCATATTTTT ATGTTTTGTT GTTTATATGC GGCCGTCTGA ACCGGAGCGA CTTACGTATA AATTAATTGC AACAGAATGG AATGATTGGG TGCGGTTATG CTTTGAAATT
i2_LQ_NR42497|c51951/f1p6/3009   ATTTTACATC TCATATTTTT ATGTTTTGTT GTTTATATGC GGCCGTCTGA ACCGGAGCGA CTTATGTATA AATTAATTGC AACAGAATGG AATGATTGGG TGCGGTTATG CTTTGAAATT
```

```
                                  1321
Bm-osm-9_Bm1711.1                 CTAACGATAG CCAGTTGTGT GTTCTTCGTT TTCTTTCAGC AGTTTAGCGA ACTAAGAACG CAAGGATTTT ATGGATATAT CAGAAATCTG AAAACGGCTC CAGCTAAAAT TGTATTTCTT
Bm-osm-9_clone                    CTAACGATAG CCAGTTGTGT GTTCTTCGTT TTCTTTCAGC AGTTTAGCGA ACTAAGAACG CAAGGATTTT ATGGATATAT CAGAAATCTG AAAACGGCTC CAGCTAAAAT TGTATTTCTT
i3_LQ_NR42497|c19820/f1p1/3020    CTAACGATAG CCAGTTGTGT GTTCTTCGTT TTCTTTCAGC AGTTTAGCGA ACTAAGAACG CAAGGATTTT ATGGATATAT CAGAAATCTG AAAACGGCTC CAGCTAAAAT TGTATTTCTT
i2_LQ_NR42497|c51951/f1p6/3009    CTAACGATAG CCAGTTGTGT GTTCTTCGTT TTCTTTCAAC AGTTTAGCGA ACTAAGAACG CAGGGATTTT ATGGATATAT CAGAAATCTG AAAACGGCTC CAGCTAAAAT TGTATTTCTT

                                  1441
Bm-osm-9_Bm1711.1                 GGTGCCAATA TATGTATTCT TATATGTGTA CCTTTTCGTA TATCGGGCAA TGTTCAAGTT GAAGAAGCTT TATTAGTCTT TTCATTACCG GGTTCTTGGA TATTTTTCCT TTTCTTTGCC
Bm-osm-9_clone                    GGTGCCAATA TATGTATTCT TATATGTGTA CCTTTTCGTA TATCGGGCAA TGTTCAAGTT GAAGAAGCTT TATTAGTCTT TTCATTACCG GGTTCTTGGA TATTTTTCCT TTTCTTTGCC
i3_LQ_NR42497|c19820/f1p1/3020    GGTGCCAATA TATGTATTCT TATATGTGTA CCTTTTCGTA TATCGGGCAA TGTTCAAGTT GAAGAAGCTT TATTAGTCTT TTCATTACCG GGTTCTTGGA TATTTTTCCT TTTCTTTGCC
i2_LQ_NR42497|c51951/f1p6/3009    GGTGCCAATA TATGTATTCT TATATGTGTA CCCTTTCGTA TATCGGGCAA TGTTCAAGTT GAAGAAGCTT TATTAGTCTT TTCATTACCG GGTTCTTGGA TATTTTTCCT TTTCTTTGCC

                                  1561
Bm-osm-9_Bm1711.1                 AGAAGTGCCA AATTAACGGG ACCATTTGTG CAAATGATTT ATAGTATGAT TGCTGGCGAT ATGATTCGCT TTGCTATCAT TTCCGCTATA TTTCTTGTCT CATTTTCGCA AGTATTTTAT
Bm-osm-9_clone                    AGAAGTGCCA AATTAACGGG ACCATTTGTG CAAATGATTT ATAGTATGAT TGCTGGCGAT ATGATTCGCT TTGCTATCAT TTCCGCTATA TTTCTTGTCT CATTTTCGCA AGTATTTTAT
i3_LQ_NR42497|c19820/f1p1/3020    AGAAGTGCCA AATTAACGGG ACCATTTGTG CAAATGATTT ATAGTATGAT TGCTGGCGAT ATGATTCGCT TTGCTATCAT TTCCGCTATA TTTCTTGTCT CATTTTCGCA AGTATTTTAT
i2_LQ_NR42497|c51951/f1p6/3009    AGAAGTGCCA AATTAACGGG ACCATTTGTG CAAATGATTT ATAGTATGAT TGCTGGCGAT ATGATTCGCT TTGCTATCAT TTCCGCTATA TTTCTTGTCT CATTTTCGCA AGTATTTTAT

                                  1681
Bm-osm-9_Bm1711.1                 TTTCTGGGAA AGGATATGCA CGTGAAGCAA GAGCTCAATC CGCTGAATCC AGACTACTGC GAAGTGAAAG GCTATGATAT CTTCACGTAT TCCTCATTTC TAGAAACATT TATTACACTG
Bm-osm-9_clone                    TTTCTGGGAA AGGATATGCA CGTGAAGCAA GAGCTCAATC CGCTGAATCC AGACTACTGC GAAGTGAAAG GCTATGATAT CTTCACGTAT TCCTCATTTC TAGAAACATT TATTACACTG
i3_LQ_NR42497|c19820/f1p1/3020    TTTCTGGGAA AGGATATGCA CGTGAAGCAA GAGCTCAATC CGCTGAATCC AGACTACTGC GAAGTGAAAG GCTATGATAT CTTCACGTAT TCCTCATTTC TAGAAACATT TATTACACTG
i2_LQ_NR42497|c51951/f1p6/3009    TTTCTGGGAA AGGATATGCA CGTGAAGCAA GAGCTCAATC CGCTGAATCC AGACTACTGC GAAGTGAAAG GCTATGATAT CTTCACGTAT TCCTCATTTC TAGAAACATT TATTACACTG

                                  1801
Bm-osm-9_Bm1711.1                 TTTCGTGCTA GCATGGGTGG CTATGATTAT GAGGAATTTT CTTGCGCTAA TTACGAAGTG TTAACAAAAA TATTATTCGT ACTTTATATG TTTATAATGC CGATCATGTT GATTAATATA
Bm-osm-9_clone                    TTTCGTGCTA GCATGGGTGG CTATGATTAT GAGGAATTTT CTTGCGCTAA TTACGAAGTG TTAACAAAAA TATTATTCGT ACTTTATATG TTTATAATGC CGATCATGTT GATTAATATA
i3_LQ_NR42497|c19820/f1p1/3020    TTTCGTGCTA GCATGGGTGG CTATGATTAT GAGGAATTTT CTTGCGCTAA TTACGAAGTG TTAACAAAAA TATTATTCGT ACTTTATATG TTTATAATGC CGATCATGTT GATTAATATA
i2_LQ_NR42497|c51951/f1p6/3009    TTTCGTGCTA GCATGGGTGG CTACGATTAT GAGGAATTTT CTTGCGCTAA TTACGAAGTG TTAACAAAAA TATTATTCGT ACTTTATATG TTTATAATGC CGATCATGTT GATTAATATA

                                  1921
Bm-osm-9_Bm1711.1                 CTGATCGCCA TGATGGGTAA TACTTATACA ACAGTCATCG CACAAGCTGA AAAAGCTTGG AGGCAACAGT ACGCGCAAAT TGTAATGGTA TTGGAAAGAT CGGTG-AAAA AGGAAAAATT
Bm-osm-9_clone                    CTGATCGCCA TGATGGGTAA TACTTATACA ACAGTCATCG CACAAGCTGA AAAAGCTTGG AGGCAACAGT ACGCGCAAAT TGTAATGGTA TTGGAAAGAT CGGTG-AAAA AGGAAAAATT
i3_LQ_NR42497|c19820/f1p1/3020    CTGATCGCCA TGATGGGTAA TACTTATACA ACAGTCATCG CACAAGCTGA AAAAGCTTGG AGGCAACAGT ACGCGCAAAT TGTAATGGTA TTGGAAAGAT CGGTGAAAAA AGGAAAAATT
i2_LQ_NR42497|c51951/f1p6/3009    CTGATCGCCA TGATGGGTAA TACTTATACA ACAGTCATCG CACAAGCTGA AAAAGCTTGG AGGCAACAGT ACGCGCAAAT TGTAATGGTA TTGGAAAGAT CGGTG-AAAA AGGAAAAATT

                                  2041
Bm-osm-9_Bm1711.1                 AGCAGCGTGT CAATTAGAGT ATAGTATTAG ACTTAATGAA GCTAATGATG CTGGTATGGA AATACGTGGT CTAATGGTCA TCAAACAGAC AAAGAAAACT CGAGCAAGAC AAAGGAAGCA
Bm-osm-9_clone                    AGCAGCGTGT CAATTAGAGT ATAGTATTAG ACTTAATGAA GCTAATGATG CTGGTATGGA AATACGTGGT CTAATGGTCA TCAAACAGAC AAAGAAAACT CGAGCAAGAC AAAGGAAGCA
i3_LQ_NR42497|c19820/f1p1/3020    AGCAGCGTGT CAATTAGAGT ATAGTATTAG ACTTAATGAA GCTAATGATG CTGGTATGGA AATACGTGGT CTAATGGTCA TCAAACAGAC AAAGAAAACT CGAGCAAGAC AAAGGAAGCA
i2_LQ_NR42497|c51951/f1p6/3009    AGCAGCATGT CAATTAGAGT ATAGTATTAG ACTTAATGAA GCTAATGATG CTGGTATGGA AATACGTGGT CTAATGGTCA TCAAACAGAC AAAGAAAACT CGAGCAAGAC AAAGGAAGCA

                                  2161
Bm-osm-9_Bm1711.1                 AGCTATCACT AATTGGAAGA CAATTGGTCG AAAAGTAATT CATACTGTGG AAAGGTTGGG TGTTGATTAT GCACAGGAAT TACTTCATTC ATACAACTGT TTAATTGATG AACCAGCTGG
Bm-osm-9_clone                    AGCTATCACT AATTGGAAGA CAATTGGTCG AAAAGTAATT CATACTGTGG AAAGGTTGGG TGTTGATTAT GCACAGGAAT TACTTCATTC ATACAACTGT TTAATTGATG AACCAGCTGG
i3_LQ_NR42497|c19820/f1p1/3020    AGCTATCACT AATTGGAAGA CAATTGGTCG AAAAGTAATT CATACTGTGG AAAGGTTGGG TGTTGATTAT GCACAGGAAT TACTTCATTC ATACAACTGT TTAATTGATG AACCAGCTGG
i2_LQ_NR42497|c51951/f1p6/3009    AGCTATCACT AACTGGAAGA CAATTGGTCG AAAAGTAATT CATACTGTGG AAAGGTTGGG TGTTGGTTAT GCACAGGAAT TACTTCATTC ATACAACTGT TTAATTGATG AACCAGCTGG

                                  2281
Bm-osm-9_Bm1711.1                 TGCTGTAATA CTTAGACGAG ATACTGTATT TCCACCACCA ACAAGGTCTA TGACAAGATC ACAAGCTCAT GAAACCTCAA CAAATACGCA AAATGAAGAA AAAATCGAAC ATTTAAATAA
Bm-osm-9_clone                    TGCTGTAATA CTTAGACGAG ATACTGTATT TCCACCACCA ACAAGGTCTA TGACAAGATC ACAAGCTCAT GAAACCTCAA CAAATACGCA AAATGAAGAA AAAATCGAAC ATTTAAATAA
i3_LQ_NR42497|c19820/f1p1/3020    TGCTGTAATA CTTAGACGAG ATACTGTATT TCCACCACCA ACAAGGTCTA TGACAAGATC ACAAGCTCAT GAAACCTCAA CAAATACGCA AAATGAAGAA AAAATCGAAC ATTTAAATAA
i2_LQ_NR42497|c51951/f1p6/3009    TGCTGTAATA CTTAGACGAG ATACTGTATT TCCACCACCA ACAAGGTCTA TGACAAGATC ACGAGCTCAT GAAACCTCAA CAAATACGCA AAATGAAGAA AAAATCGAAC ATTTAAATAA

                                  2401
Bm-osm-9_Bm1711.1                 TATCTCAATC GAGAAAGAAA TAATCGTAAA TAATTGTTCC AACCAAACGT CTCTCCAATG TCAGTCACCG CGAGATTTTA ---------- ---------- ---------- ----------
Bm-osm-9_clone                    TATCTCAATC GAGAAAGAAA TAATCGTAAA TAATTGTTCC AACCAAACGT CTCTCCAATG TCAGTCACCG CGAGATTTTA AATTAATAAC AACCAAACAA AGCACAACTA TGATTCTGGA
i3_LQ_NR42497|c19820/f1p1/3020    TATCTCAATC GAGAAAGAAA TAATCGTAAA TAATTGTTCC AACCAAACGT CTCTCCAATG TCAGTCACCG CGAGATTTTA AATTAATAAC AACCAAACAA AGCACAACTA TGATTCTGGA
i2_LQ_NR42497|c51951/f1p6/3009    TATCTCAATC GAGAAAGAAA TAATCGTAAA TAATTGCTCC AACCAAACGT CTCTCCAATG TCAGTCACCG CGAGATTTTA AATTAATAAC AACCGAACAA AGCACAACTA TGATTCTGGA

                                  2521
Bm-osm-9_Bm1711.1                 -AAGTTGCCA ATGCGGAAAA CGGCACGAGG ACATCAGTTG GTTCCATCGT TAGACATACC GAATATGCCA GCTGCTGGAA CACCACCACG AGCAGTATCA CCGAGGATTC GAAAAGATAT
Bm-osm-9_clone                    GAAGTTGCCA ATGCGGAAAA CGGCACGAGG ACATCAGTTG GTTCCATCGT TAGACATACC GAATATGCCA GCTGCTGGAA CACCACCACG AGCAGTATCA CCGAGGATTC GAAAAGATAT
i3_LQ_NR42497|c19820/f1p1/3020    GAAGTTGCCA ATGCGGAAAA CGGCACGAGG ACATCAGTTG GTTCCATCGT TAGACATACC GAATATGCCA GCTGCTGGAA CACCACCACG AGCAGTATCA CCGAGGATTC GAAAAGATAT
i2_LQ_NR42497|c51951/f1p6/3009    GAAGTTGCCA ATGCGGAAAA CGGCACGAGG ACATCAGTTG GTTCCATCGT TAGACATACC GAATATGCCA GCTGCTGGAA CACCACCACG AGCAATATCA CCGATGATTC GAAAAGATAT
```

```
                                   2641
Bm-osm-9_Bm1711.1                  GTTTCGGCGC AAAGACAGTT TC-TGTGCGA GTTCATCATC TGCCAGCTCC AGTACGCAGG TTTGACATCA AGGATAAATA ATACGTCAAA AAATGTAACA ATTGCAGTCA ACTTCAATTT
Bm-osm-9_clone                     GTTTCGGCGC AAAGACAGTT TC-TGTGCGA GTTCATCATC TGCCAGCTCC AGTACGCAGG TTTGACATCA AGGATAAATA ATACGTCAAA AAATGTAACA ATTGCAGTCA ACTTCAATTT
i3_LQ_NR42497|c19820/f1p1/3020     GTTTCGGCGC AAAGACAGTT TCTTGTGCGA GTTCATCATC TGCCAGCTCC AGTACGCAGG TTTGACATCA AGGATAAATA ATACGTCAAA AAATGTAACA ATTGCAGTCA ACTTCAATTT
i2_LQ_NR42497|c51951/f1p6/3009     GTTTCGGCGC AAAGACAGTT TC-TGTGCGA GTTCATCATC TGCCAGCTCC AGTACGCAGG TTTGACATCA AGGATAAATA ATACGTCAAA AAATGTAACA GTTGCAGTCA ACTTCAATTT

                                   2761
Bm-osm-9_Bm1711.1                  CAGTGGCTGA ---------- ---------- ---------- ---------- ---------- ---------- ---------- ---------- ----
Bm-osm-9_clone                     CAGTGGCTGA ---------- ---------- ---------- ---------- ---------- ---------- ---------- ---------- ----
i3_LQ_NR42497|c19820/f1p1/3020     CAGTGGCTGA ATTTATGATG TGTTCTCAAA ATTGTTTTAA TTAC-ATTCT TCTAATGCCA GTTTGTTTGC CTTATCAATA AAAATTTACG ----
i2_LQ_NR42497|c51951/f1p6/3009     CAGTGGCTGA ATTTATGATG TGTTCTCAAA ATTGTTTTAA TTACAATTCT TCTAATGCCA G-TTGTTTGC CTGATC-ATA AAAATTTACG AGAC
```
