# Supplementary material for: Genetic and functional diversification of chemosensory pathway receptors in mosquito-borne filarial nematodes
Source: PLoS Biol. 2020 Jun 8;18(6):e3000723. doi: 10.1371/journal.pbio.3000723 (PMC7302863; doi:10.1371/journal.pbio.3000723)
Supplement: S9 Fig — The mispredicted splice donor in the predicted gene model (Bm-tax-4_Bm7343.1), which led to a 7-aa deletion, can be seen on the line starting with amino acid 131. (PDF) [file pbio.3000723.s014.pdf]

```
                    1
Ce-tax-4_ZC84.2.1   MSTAEPAPDP TNPSTSGLAP TTNGIGSPPP TASAATKFSI LTKFLRR--- ---------- --KNQVHTTT AQQNEFMQKY MPNGNSNAVQ PAATGGQPAS SDGGSAIEVP PPKESYAVRI RKYLANYTQD
Bm-tax-4_Bm7343.1   MFSK--SHDD T--YENHLKK REN-----LP NASAMKNISN NDSFQKRHKS LRSNKICNIN EEENQAPSTV TNTPQLNSID SNKSCEKAKQ SLIETSAPTS LTSKQPATEA PSATTHETKW QYLLNKWVLD
Bm-tax-4_clone      MFSK--SHDD T--YENHLKK REN-----LP NASAMKNISN NDSFQKRHKS LRSNKICNIN EEENQAPSTV TNTPQLNSID SNKSCEKAKQ SLIETSAPTS LTSKQPATEA PSATTHETKW QYLLNKWVLD

                  131
Ce-tax-4_ZC84.2.1   PSTDNFYYWT CVVTVAYIYN LLFVIARQVF NDLIGPSSQS LCRFYNGTLN STTQVECTYN MLTNMKEMPT YSQYPDLGWS KYWHFRMLWV FFDLLMDCVY LIDTFLNYRM GYMDQGLVVR EAEKVTKAYW
Bm-tax-4_Bm7343.1   TKDEFYYYWL SIVSCAFTYN LIVVIGKLFF ---------- ---------- ---------- -------LLS RSVFNDLAYG YYW---IAWL LVDFIMDITY VLDMFVRSRT GFLEQGLLVR DISRISKLYL
Bm-tax-4_clone      TKDEFYYYWL SIVSCAFTYN LIVVI----- ---------- ---------- ---------- ---------A RSVFNDLAYG YYW---IAWL LVDFIMDITY VLDMFVRSRT GFLEQGLLVR DISRISKLYL

                  261
Ce-tax-4_ZC84.2.1   QSKQYRIDGI SLIPLDYILG WPIPYINWRG LPILRLNRLI RYKRVRNCLE RTETRSSMPN AFRVVVVVWY IVIIIHWNAC LYFWISEWIG LGTDAWVYGH LNKQSLPDDI TDTLLRRYVY SFYWSTLILT
Bm-tax-4_Bm7343.1   KSLQFKLDII SVLPFDLILS ----FIFQRS IPYLRFNRII RYPRFSDFVD RTETRSSMPN AFRIFCVIVN IIVIIHWNAC IYFFISEMIG LGSDGWVYGP LNKQSLPDGV EDTLVRRYIY SFYWSTLILT
Bm-tax-4_clone      KSLQFKLDII SVLPFDLILS ----LIFQRS IPYLRFNRII RYPRFSDFVD RTETRSSMPN AFRIFCVIVN IIVIIHWNAC IYFFISEMIG LGSDGWVYGP LNKQSLPDGV EDTLVRRYIY SFYWSTLILT

                  391
Ce-tax-4_ZC84.2.1   TIGEVPSPVR NIEYAFVTLD LMCGVLIFAT IVGNVGSMIS NMSAARTEFQ NKMDGIKQYM ELRKVSKQLE IRVIKWFDYL WTNKQSLSDQ QVLKVLPDKL QAEIAMQVHF ETLRKVRIFQ DCEAGLLAEL
Bm-tax-4_Bm7343.1   TIGEVPSPKR NIEFLFVIMD LMCGVLIFAT IVGNVGSAIS NMSLARTKFQ NKMDGIKQYM KLRKVNKELE TRVMKWFDYL WEHKQSLSDQ RVLKVLPDKL QTEIAMQVHY ETLRRVRIFQ DCEAGLLAEL
Bm-tax-4_clone      TIGEVPSPKR NIEFLFVIMD LMCGVLIFAT IVGNVGSAIS NMSLARTKFQ NKMDGIKQYM KLRKVNKELE TRVMKWFDYL WEHKQSLSDQ RVLKVLPDKL QTEIAMQVHY ETLRRVRIFQ DCEAGLLAEL

                  521
Ce-tax-4_ZC84.2.1   VLKLQLQVFS PGDFICKKGD IGREMYIVKR GRLQVVDDDG KKVFVTLQEG SVFGELSILN IAGSKNGNRR TANVRSVGYT DLFVLSKTDL WNALREYPDA RKLLLAKGRE ILKKDNLLDE NAPEEQKTVE
Bm-tax-4_Bm7343.1   VLKLQQQIFS PGDYICKKGD IGREMYIVKR GKLQVVADDG IKVFATLQEG AVFGELSILN IAGSKNGNRR TANVRSVGYT DLFALNKNDL WTALKEYPDA RKLLIAKGRE ILRKDGLLDE DAPEEQMTAE
Bm-tax-4_clone      VLKLQQQIFS PGDYICKKGD IGREMYIVKR GKLQVVADDG IKVFATLQEG AVFGELSILN IAGSKNGNRR TANVRSVGYT DLFALNKNDL WTALKEYPDA RKLLIAKGRE ILRKDGLLDE DAPEEQMTAE

                  651
Ce-tax-4_ZC84.2.1   EIAEHLNNAV KVLQTRMARL IVEHSSTEGK LMKRIEMLEK HLSRYKALAR RQKTMHGVSI DGGDISTDGV DERVRPPRLR QTKTIDLPTG TESESLLK
Bm-tax-4_Bm7343.1   EMAKNLQNTL KIMQTKMARF AAEFSSVKTK LLARIEYLET QLAKYQI--- ---------- --NDNSTSSN D--------- -----DYQMI CDS-----
Bm-tax-4_clone      EMAKNLQNTL KIMQTKMARF AAEFSSVKTK LLARIEYLET QLAKYQI--- ---------- --NDNSTSSN D--------- -----DYQMI CDS-----
```
