# Supplementary material for: Genetic and functional diversification of chemosensory pathway receptors in mosquito-borne filarial nematodes
Source: PLoS Biol. 2020 Jun 8;18(6):e3000723. doi: 10.1371/journal.pbio.3000723 (PMC7302863; doi:10.1371/journal.pbio.3000723)
Supplement: S10 Fig — All transgenic strains had detectable RNA levels of the transgenes. Raw data can be found at https://github.com/zamanianlab/BrugiaChemo-ms. ND, not determined; qPCR, quantitative PCR. (PDF) [file pbio.3000723.s015.pdf]

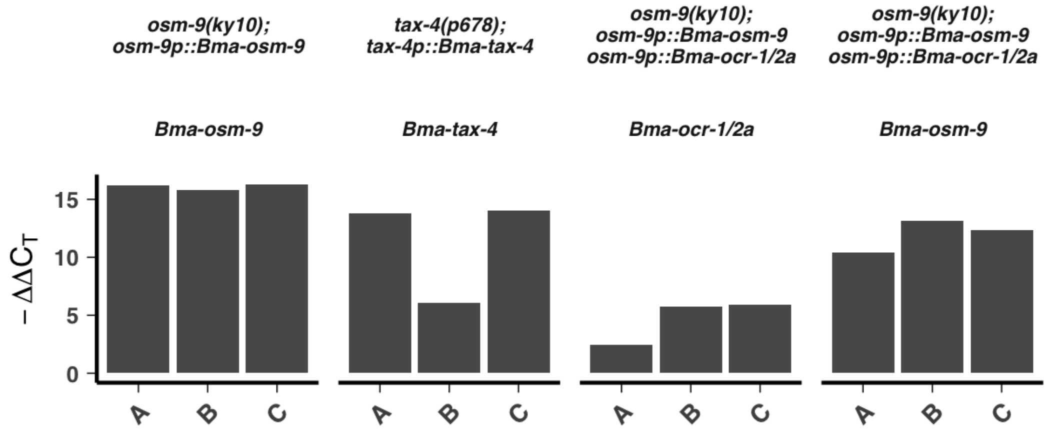

Raw C_T values for Transgenes

	Target			
**Strains**	***Y45F10D.4***	***Bma-osm-9***	***Bma-ocr-1/2a***	***Bma-tax-4***
**Controls**				
N2	24.18	ND	ND	ND
osm-9(ky10)	23.93	ND	ND	
tax-4(p678)	23.54			ND
osm-9(ky10); osm-9p::Bma-osm-9				
A	24	23.88		
B	24.14	24.44		
C	23.72	23.47		
osm-9(ky10); osm-9p::Bma-osm-9; osm-9p::Bma-ocr-1/2a				
A	23.56	29.19	37.20	
B	23.96	26.91	34.30	
C	23.79	27.51	33.90	
tax-4(p678); tax-4p::Bma-tax-4				
A	24.3			26.98
B	23.99			34.38
C	23.72			26.13

Note: ND = Not Detected
