# Supplementary material for: Genetic and functional diversification of chemosensory pathway receptors in mosquito-borne filarial nematodes
Source: PLoS Biol. 2020 Jun 8;18(6):e3000723. doi: 10.1371/journal.pbio.3000723 (PMC7302863; doi:10.1371/journal.pbio.3000723)
Supplement: S4 File — (PDF) [file pbio.3000723.s021.pdf]

Cloning, dsRNA Synthesis, and qPCR Protocols

Brugia malayi Gene Cloning

Bm-osm-9 expression cloning

ORF cloning:

Forward primer: ATGGGACAACTAAAGAGCAAAA
Reverse primer: TCAGCCACTGAAATTGAA

Template: Adult female *Brugia malayi* cDNA synthesized with oligo(dT) primers

Stage	Temperature	Length	Cycles
Melt	98°C	30 s	
Melt	98°C	10 s	
Anneal	58°C	30 s	40
Extend	65°C	3 min	
Extend	65°C	5 min	

Primers for Sanger sequencing:

M13F
M13R
TACAATATGGCGCTGATCCA
CGTATATCGGGCAATGTTCA

Promoter cloning:

Forward primer: GGTGGT-TCTAGA-TTTTAAAAAGGTTTTTGAGAATCAG
Reverse primer: GGTGGT-GGATCC-GTTTGGTTTCTGAAAAAATTGG

Template: *C. elegans* N2 genomic DNA

Stage	Temperature	Length	Cycles
Melt	98°C	30 s	
Melt	98°C	10 s	
Anneal	63°C	30 s	5
Extend	65°C	1:40 min	
Melt	98°C	10 s	
Anneal/Extend	65°C	2:10 min	30
Extend	65°C	5 min	

Preparation for HiFi Assembly

Ce-osm-9 promoter

Forward primer: TGCATGCCTGCAGGTCGACTCTAGA-TTTTAAAAAGGTTTTTGAGAATCAG
Reverse primer: GTTGTCCCAT-GGATCC-GTTTGGTTTCTGAAAAAATTG

Template: *Ce-osm-9* promoter in pGEM-T

Stage	Temperature	Length	Cycles
Melt	98°C	30 s	
Melt	98°C	10 s	
Anneal	59°C	30 s	35
Extend	72°C	1:30 min	
Extend	65°C	5 min	

Bm-osm-9 ORF

Forward primer: GAAACCAAACGGATCC-ATGGGACAACTAAAGAGC
Reverse primer: AGCGACCGGCGCTCAGTTG-GAATTC-AGCCACTGAAATTGAAG

Template: *Bm-osm-9* ORF in pGEM-T

Stage	Temperature	Length	Cycles
Melt	98°C	30 s	
Melt	98°C	10 s	
Anneal	67°C	30 s	35
Extend	72°C	1:30 min	
Extend	65°C	5 min	

HiFi Assembly Parameters

Vector amount: 0.023 pmol
Promoter amount: 0.046 pmol
ORF amount: 0.046 pmol

Primers for Sanger sequencing:

CGATGGATACGCTAACAACTTGG
ACTAAGAAGGCGGAGTTGGC
TACAATATGGCGCTGATCCA
CGTATATCGGGCAATGTTCA
GAGCACAGGGAGAAAGAGCA

Bm-ocr-1/2a expression cloning

ORF cloning:

Forward primer: ATGGGAAATGTGGAATCGTCG
Reverse primer: TTGTCGATGCCACAGTGAAC

Template: Adult female *Brugia malayi* cDNA synthesized with random hexamer primers

Stage	Temperature	Length	Cycles
Melt	98°C	30 s	
Melt	98°C	10 s	
Anneal	58°C	30 s	40
Extend	65°C	3 min	
Extend	65°C	5 min	

Primers for Sanger sequencing:

M13F
M13R
TCTACTTCGACATGGTGCTGA
GAGCACATCAAACGAAATGGT

Preparation for HiFi Assembly

***Bm-ocr-1/2a* ORF**

Forward primer: ATTTTTTCAGAAACCAAAC-GGATCC-ATGGGAAATGTGGAATCG
Reverse primer: AGCGACCGGCGCTCAGTTG-GAATTC-TATTCTCCTAGAACCTTTAATC

Template: *Bm-ocr-1/2a* ORF in pGEM-T

Stage	Temperature	Length	Cycles
Melt	98°C	30 s	
Melt	98°C	10 s	
Anneal	58°C	30 s	35
Extend	72°C	1:30 min	
Extend	72°C	5 min	

HiFi Assembly Parameters

Vector amount: 0.023 pmol
Promoter amount: 0.046 pmol
ORF amount: 0.046 pmol

Primers for Sanger sequencing:

ACTAAGAAGGCGGAGTTGGC
TCTACTTCGACATGGTGCTGA
GAGCACATCAAACGAAATGGT
GAGCACAGGGAGAAAGAGCA

Bm-tax-4 expression cloning

ORF cloning:

Forward primer: ATGTTCTCTAAAAGTCATGATGA
Reverse primer: TTAACTATCACATATCATCTGATAATC

Template: Adult female *Brugia malayi* cDNA synthesized with oligo(dT) primers

Stage	Temperature	Length	Cycles
Melt	98°C	30 s	
Melt	98°C	10 s	
Anneal	58°C	30 s	40
Extend	65°C	1:30 min	
Extend	65°C	5 min	

Primers for Sanger sequencing:

M13F
M13R
CCTGAAAAATGCGTACTCTTCTAA

Promoter cloning:

Forward primer: GGTGGT-TCTAGA-ACCATCACTGAAGGGTGAGC
Reverse primer: GGTGGT-GGATCC-TCTTGA-AACATAATTAAATTTTGAGAATGATAG

Template: *C. elegans* N2 genomic DNA

Stage	Temperature	Length	Cycles
Melt	98°C	30 s	
Melt	98°C	10 s	
Anneal	64°C	30 s	5
Extend	65°C	1:40 min	
Melt	98°C	10 s	
Anneal/Extend	65°C	2:10 min	30
Extend	65°C	5 min	

Preparation for HiFi Assembly

Ce-tax-4 promoter

Forward primer: TGCATGCCTGCAGGTCGACTCTAGAGGTGGTTCTAGA-ACCATCACTG
Reverse primer: TAGAGAACA-TGGTGGT-GGATCC-TCTTGAAAC

Template: *Ce-tax-4* promoter in pGEM-T

Stage	Temperature	Length	Cycles
Melt	98°C	30 s	
Melt	98°C	10 s	
Anneal	65°C	30 s	35
Extend	72°C	1:30 min	
Extend	72°C	5 min	

Bm-tax-4 ORF

Forward primer: ATCCACCACC-ATGTTCTCTAAAAGTCATGATGATAC
Reverse primer: AGCGACCGGCGCTCAGTTGGAATTC-TTAACTATCACATATCATCTGATAATC

Template: *Bm-tax-4* ORF in pGEM-T

Stage	Temperature	Length	Cycles
Melt	98°C	30 s	
Melt	98°C	10 s	
Anneal	59°C	30 s	35
Extend	72°C	1:30 min	
Extend	72°C	5 min	

HiFi Assembly Parameters

Vector amount: 0.023 pmol
Promoter amount: 0.046 pmol
ORF amount: 0.046 pmol

Primers for Sanger sequencing:

CGATGGATACGCTAACAACTTGG
TAGAGAACATGGTGGTGGATCCTCTTGAAAC
CGTAAATAGGGTATTGATCGTTGA
CCTGAAAAAATGCGTACTCTTCTAA
GAGCACAGGGAGAAAGAGCA

Caenorhabditis elegans Gene Cloning

Ce-osm-9 expression cloning

Preparation for HiFi Assembly

Forward primer: ATTTTTTCAGAAACCAAAC-GGATCC-ATGGGCGGTGGAAGTTCG
Reverse primer: AGCGACCGGCGCTCAGTTG-GAATTC-TCATTCGCTTTTGTCATTTGTCG

Template: Psra-6::osm-9(cDNA)::sl2::CFP vector from the Shawn Xu lab (Neuron, Wang 2016)

Stage	Temperature	Length	Cycles
Melt	98°C	30 s	
Melt	98°C	10 s	
Anneal	65°C	30 s	35
Extend	72°C	1:10 min	
Extend	72°C	2 min	

HiFi Assembly Parameters

Vector amount: 0.015 pmol
ORF amount: 0.046 pmol

Primers for Sanger sequencing:

ACTAAGAAGGCGGAGTTGGC
GCATTTGCCGCTTGTTTTGG
CGGCGCCAACAACCATTG
GAGCACAGGGAGAAAGAGCA

***Ce-tax-4* expression cloning**

Preparation for HiFi Assembly

Forward primer: ATTTAATTATGTTTCAAGAG-GGATCC-ATGTCAACGGCGGAACCTG
Reverse primer: ACCGGCGCTCAGTTGGAATT-GAATTC-CTATTTGAGCAAGGATTCAGATTCAGTTC

Template: pEM04 (gcy-36p::tax-4::sl2::GFP) vector from the Cornelia Bargmann lab (Nature, Macosko 2009)

Stage	Temperature	Length	Cycles
Melt	98°C	30 s	
Melt	98°C	10 s	
Anneal	66°C	30 s	35
Extend	72°C	1:15 min	
Extend	72°C	2 min	

HiFi Assembly Parameters

Vector amount: 0.012 pmol
ORF amount: 0.024 pmol

Primers for Sanger sequencing:

TTTGTGGTTCAGGCTGCTCA

TCAACGATCTCATTGGACCATCT
TCCGGTGCATTTTCATCCAA
GAGCACAGGGAGAAAGAGCA

Replacing *unc-54* 3' UTR with *osm-9* 3' UTR

Bm-osm-9 **expression cloning**

Preparation for HiFi Assembly

osm-9 **3' UTR cloning:**

Forward primer: AACTTCAATTTCAGTGGCTGAATTC-GAACTTTTTTCTTCTAATTTTTTGAAAAC
Reverse primer: CGCGCGAGACGAAAGGGCCCGTACG-AGTAAATTTGGCAATTTCTG

Template: *C. elegans* N2 genomic DNA

Stage	Temperature	Length	Cycles
Melt	98°C	30 s	
Melt	98°C	10 s	
Anneal	56°C	30 s	35
Extend	72°C	2 min	
Extend	72°C	2 min	

HiFi Assembly Parameters

Vector amount: 0.0115 pmol
ORF amount: 0.0231 pmol

Primers for Sanger sequencing:

GTTTCGGCGCAAAGACAGTT
TTAAAGGGCGCACTCTTCCG
TCCCGAAAGTTGATCTCCGA
AACTTTTGGAGGCGGGTGAG
CATAGTTAAGCCAGCCCCGA

Ce-osm-9 **expression cloning**

Preparation for HiFi Assembly

osm-9 **3' UTR cloning:**

Forward primer: ACAAAAGCGAATGAGAATTCGAACTTTTTTCTTC-TAATTTTTTGAAAACT
Reverse primer: GAGACGAAAGGGCCCGTACGAGTAAATTTG-GCAATTTCTGGC

Template: *C. elegans* N2 genomic DNA

Stage	Temperature	Length	Cycles
Melt	98°C	30 s	
Melt	98°C	10 s	
Anneal	60°C	30 s	35
Extend	72°C	2 min	
Extend	72°C	2 min	

***Ce-osm-9* backbone cloning:**

Forward primer: CAGAAATTGCCAAATTTACTCGTACGGGCC-CTTTCGTCTC
Reverse primer: AAATTAGAAGAAAAAAGTTCGAATTCTCATTCGCTTTTGT-CATTTG

Template: mazEx18

Stage	Temperature	Length	Cycles
Melt	98°C	30 s	
Melt	98°C	10 s	
Anneal	64°C	30 s	35
Extend	72°C	3:30 min	
Extend	72°C	2 min	

HiFi Assembly Parameters

Vector amount: 0.0114 pmol
ORF amount: 0.0228 pmol

Primers for Sanger sequencing:

CCGGCTTCGTTTGATCAGAG
TTAAAGGGCGCACTCTTCCG
TCCCGAAAGTTGATCTCCGA
AACTTTTGGAGGCGGGTGAG
CATAGTTAAGCCAGCCCCGA

dsRNA Synthesis

Bm-osm-9 Target

Forward primer + T7: CGATGTTAATACGACTCACTATAGGG-CACCATTGACGCTTGCAACA
Reverse primer + T7: CGATGTTAATACGACTCACTATAGGG-ACCGCACCCAATCATTCCAT

Template: *Bm-osm-9* Expression Plasmid

Stage	Temperature	Length	Cycles
Melt	98°C	30 s	
Melt	98°C	10 s	
Anneal	59°C	30 s	35
Extend	72°C	1:30 min	
Extend	65°C	5 min	

Bm-tax-4 **Target**

Forward primer + T7: TAATACGACTCACTATAGGGAGA-TTCCGGATAAATTGCAAACAG
Reverse primer + T7: TAATACGACTCACTATAGGGAGA-CAAATCGAGCCATTTTGGTT

Template: *Bm-tax-4* Expression Plasmid

Stage	Temperature	Length	Cycles
Melt	98°C	30 s	
Melt	98°C	10 s	
Anneal	59°C	30 s	35
Extend	72°C	1:30 min	
Extend	65°C	5 min	

qPCR Primers

Target	Sequence
Bm-GAPDH	TTTCTGCAGAGGGAGGCAAG
Bm-GAPDH	TCAGCGGGATCTTTGCTGTT
Bm-osm-9	CCCGCTGATCCAAACATTG
Bm-osm-9	TGCACTACACGTCATATCACTG
Bm-tax-4	TTGGCTCAGATGGTTGGGTC
Bm-tax-4	TTGGACTTGGCACTTCACCG
Bm-ocr-1/2a	ATCTACGTTGGCAAGGCGAT
Bm-ocr-1/2a	CCACTGTCGTTTCCACTCAG
Y45F10D.4	GTCGCTTCAAATCAGTTCAGC
Y45F10D.4	GTTCTTGTCAAGTGATCCGACA
