# Supplementary material for: Genetic and functional diversification of chemosensory pathway receptors in mosquito-borne filarial nematodes
Source: PLoS Biol. 2020 Jun 8;18(6):e3000723. doi: 10.1371/journal.pbio.3000723 (PMC7302863; doi:10.1371/journal.pbio.3000723)
Supplement: Attachment — Submitted filename: Reviewer_Responses.pdf [file pbio.3000723.s022.pdf]

Reviewer 1

Major Issues:

1. *The authors performed a great intra-mosquito RNAi experiment. I appreciated the disclosure that while they attempted to confirm knockdown by qPCR they were unable to measure expression for reasons they provided. While a nonspecific RNAi control was good to include, I think the authors should include knockdown of a chemoreceptor not thought to be involved in this process, as is a common practice in C. elegans research. This would illustrate that the behavior is specific to the knockdown of osm-9 and tax-4 rather than successful knockdown of a channel receptor in the microfilariae.*

   I appreciated that the authors included a nice control when examining the effect of treating with nicotinamide by measuring the relative expression of Bp-osm-9 in L3s under different conditions up to 4 hours after being removed from the mosquito host. In the subsequent paragraph of the results the authors discuss the loss of chemotactic response of nematodes that were maintained overnight under standard culture conditions. I also appreciated their interpretation of the loss of chemotaxis toward serum after an overnight culture.

   Thank you for these thoughtful comments. The standard in the field (Kashyap et al. 2019, PLoS Pathogens, Verma et al. 2017, PNAS) has been to use non-specific dsRNAs as controls, partly given lack of confidence about gene annotations and genome quality in *Brugia* spp.. In our case, we would also have to contend with the difficulty in detecting expression differences in unannotated chemoreceptors that are likely to be expressed in a single or a handful of neurons. For this study, we chose to use lacZ dsRNA as it has been used along with other non-specific dsRNAs (e.g., GFP) as a control in both larval and adult RNAi experiments. No phenotypes have been attributed to these control triggers and they have been used as controls to identify specific phenotypes mediated by genes across diverse pathways (e.g., cathepsins and nicotinic receptors). As we grow these experiments in new directions, we expect to clone additional genes in relevant pathways that can potentially serve as additional controls.

Minor Issues:

1. *The figures should be reassessed for correct numbering. The results section on "Treatment with a nematode TRPV agonist inhibits chemoattraction…" erroneously refers to incorrect panels of figure 4. The current reference to Figure 4A should refer to Figure 4B. The current reference to Figure 4B should probably be moved to the previous sentence and a new reference to Figure 4C should be put in its place. The current reference to Figure 4C should be changed to figure 4D. The current references to Figure 4D should be switched to reference Figure 4E.*

Thank you for catching these labelling errors; they have been corrected (lines 204-231).

There are also some modifications that could be made to the figures to facilitate each of interpretation. One suggestion is that the authors could include a gene name title for Figure 3 panels C-F (e.g. osm-9 for panel C) that would allow readers to immediately identify the gene trees without having to bounce between the legend and the figure. The little diagram regarding the chemotaxis assay setup used in figure 4 and figure 7 should be explained in the legend. I realize that it is explained in the methods section, but it should also be explained in the legends of these figures.

These are helpful suggestions to ease reading and have been implemented.

2. *The references need to be evaluated and corrected. For example, there are many references with "[internet]" in them. For example, references 5, 18, 21, 34, 46, 49, 76, and 77 have this error. There are also several places in the manuscript where instead of using numbers the last names of authors are used. This occurs in several places including in the 2nd paragraph of the introduction and at the end of the 4th paragraph of the introduction, and again in the last paragraph of the discussion. PLoS formatting style requires that numbers be used throughout the text rather than author names.*

   We have re-rendered the bibliography to meet the PLoS formatting style requirements.

3. *In the 2nd page of the results section the authors discuss how their pan-phylum analysis revealed a significant correlation between chemoreceptor gene count and the presence and nature of free-living or environmental stages of each nematode species life cycle. While the analysis in this paper includes more genomes than previous studies, and is more thorough and quantitative in their analysis, other researchers have previously reported this finding and have hypothesized that species with fewer environmental stages have a more compact repertoire of chemoreceptors. That the current study supports this previously reported hypothesis is interesting, and these authors should cite the previous studies [1, 2].*

   We have added these references and a sentence noting these previous observations. We've also added an additional reference to Desjardins et al 2013, which made a similar observation (lines 137-139).

References:

1. Srinivasan, J., Dillman, A.R., Macchietto, M.G., Heikkinen, L., Lakso, M., Fracchia, K.M., Antoshechkin, I., Mortazavi, A., Wong, G., and Sternberg, P.W. (2013). The draft genome and transcriptome of Panagrellus redivivus are shaped by the harsh demands of a free-living lifestyle. Genetics 193, 1279-1295.

2. Thomas, J.H., and Robertson, H.M. (2008). The Caenorhabditis chemoreceptor gene families. BMC biology 6, 42.

Reviewer 2

Minor issues:

1. *Most importantly, the failure to detect functional expression of the two Brugia proteins in complementation experiments in C. elegans is not really conclusive. The analysis would be strengthen by the inclusion of the respective positive control (the C. elegans gene) to demonstrate success of the technique in the authors laboratory.*

   Thank you for this critique. We took the reviewer's advice and performed the positive control which illuminated the need for alternative *cis*-regulatory elements for expression in the proper neurons. This section has been greatly expanded with new transgenic strains, new assays, new figures, and modified conclusions (lines 320-336).

2. *The text is sprinkled with references cited by name instead of number, an oversight that needs to be cleaned up (renumbering may be necessary). Please also ensure appropriate italicization in the journal titles.*

   Thank you for catching this. We have re-rendered the bibliography to meet the PLoS formatting style requirements.

3. *Reference 18 seems to incomplete*

   We have re-rendered the bibliography to fix citation issues.

4. *Page 14: How do you know that NAM was restricted to the midgut and did not distribute throughout the mosquito body?*

   This is a fair point. We have added a qualifier ("presumably", line 273) to acknowledge that NAM from the blood meal may have distributed beyond the midgut - though we still expect parasite exposure to NAM to be reduced.

5. *Page 19: How many 1 mm segments were pooled for these analyses? Were independent biological replicates analyzed?*

   1 mm head and tail regions from 18 individual adult males and 19 adult females females were pooled to achieve sufficient sequencing coverage of these lowly-expressed receptors. Although these adults came from different jird (hamster) infections,

replicate-specific data were lost through pooling. This information has now been added to the methods section (lines 510-511).

Reviewer 3

1. *Pg. 1, line 11 of main text: It might be worth noting here that there are also constraints on the use of ivermectin in onchocerciasis patients where the risk of Onchocerca volvulus and Loa loa co-infection is present.*

   Thank you for catching this. We have added that both ivermectin and DEC are contraindicated in cases of coinfection (line 48).

2. *I can absolutely understand the difficulty in confirming knockdown of Bm-osm-9- and Bm-tax-4-specific messages within the experimental context of the RNAi studies, given the small numbers of L3 present and the imperative to recover sufficient numbers of them for chemotaxis assay. However, I still think it is crucial to provide data, even data gathered outside of the experimental context, that confirms knockdown efficiency and specificity of the protocol used. As a second-best approach, couldn't some "in squito" dsRNA exposures, without the constraint of gathering L3 for chemotaxis assay, be dedicated to gathering this important control data for each target gene and the control lacZ?*

   In our attempts to quantify knock-down we did in fact use RNA from parasites that were used from experiments (e.g. they were removed from assay plates) as well as from parasites that were simply collected from dsRNA-exposed infected mosquitoes. The bottleneck in our case is the number of injections that we are able to perform for a given replicate and is further complicated by variable parasite burdens and some mosquito death due to injection.

3. *Similarly, the negative findings of the heterologous complementation studies would be bolstered by inclusion of a positive control involving C. elegans osm-9(ky10) and Tax4(p768) mutants transformed with homologous (C. elegans-derived) coding sequences under their respective promoters. Given the negative results of the heterologous rescue attempts, it seems essential to prove that rescue with homologous sequences is possible before concluding divergence of function of the parasite elements.*

   Please see our response to reviewer 2, which incorporates these critiques and greatly strengthens the interpretation of the rescue experiments.

4. *The paper would be improved by a more detailed account in Materials and Methods of how the transgenic lines used in the heterologous complementation studies were derived. For example, were several independently derived lines of C. elegans*

transformed with the parasite sequences assessed for rescuing ability? Given the stochastic process of forming episomal transgene arrays in C. elegans, it is generally considered essential to base conclusions on studies of at least three independently derived lines. This shouldn't be burdensome as independent lines can be derived from hermaphrodites that are singled after microinjection with their progeny grown in isolation.

We did indeed create and maintain 3 independent lines for each transgenic strain, and worms from each line were pooled during washes prior to chemotaxis experiments. These details have been added to the material and methods section (lines 443-444; 669).

Minor issues:

1. *Pg. 4 of main text, line 26: suggest deleting "worms". It's redundant.*
2. *Pg. 4, line 31: suggest "repelled" instead of "repulsed".*
3. *Pg. 4, line 36: suggest "…specific defect in chemotaxis…".*
4. *Pg. 4, line 37: suggest inserting "that" after "ensure".*
5. *Pg. 5, lines 1-3: suggest moving the reference "(Figure 4D)" in line 3 to come after "extraction" in line 1 and adding a reference to Figure 4E after "serum-free media" in line 3.*
6. *Pg. 8, line 18 and elsewhere in the paper: the word "data" is plural, so make it "Our data suggest". Same in line 25 and several other places (eg. caption to Fig. 11).*

Thank you for your careful reading. All of these changes have been implemented (lines 206, 211, 216, 218, 225-228).

Reviewer 4

Major issues:

1. *In Figure 1c, I'm not clear on how it was determined which species have more environmental exposure for some of the categories. For example, among the EPNs, skin-penetrating nematodes, and passively ingested nematodes such as H. contortus, why are some considered to have more environmental exposure than others, when these worms have a similar third-larval stage in the environment. A bit more justification is needed for how some of the worms were ordered within each subcategory.*

We agree that there is some unclarity regarding the way the plot was designed. First, there was random jitter added to the x-axis to aid interpretation by decreasing point and label overlap; this has been made clear in the figure caption. Additionally, we have added a supporting file (S2 File) with the list of species included in the figure, the

category to which they were assigned, and a brief justification of the assignment based on the parasite's life cycle.

2. *Are all the parasite genomes equally complete? Could some of the differences in numbers of predicted receptors be due to the quality of the available genomes?*

   The various nematode parasite genomes recently released through the International Helminth Genomes Initiative have different levels of completeness. From each clade or species, where multiple genomes are available, we selected the one with highest contiguity. To ensure that genome quality was not a confounding factor in our analyses, we have added a supporting figure (S2 Figure) showing that genome contiguity does not correlate with chemoreceptor counts across analyzed genomes. To aid in transparency and in the spirit of open science, we have created an interactive plot, hosted at https://zamanianlab.shinyapps.io/ChemoR/, which allows the user to select and deselect categories, view transformed and untransformed data, and alter the variable plotted on the y-axis. The relevant statistics will also be recalculated based on the user's input. Additionally, the app also provides a portal for download of all the chemoreceptor data, which includes species name, chemoreceptor transcript ID (WormBase ParaSite nomenclature), family and superfamily categorization, and the amino acid sequence.

3. *In Figure 2a and 2b, the colors that indicate the superfamilies are a bit confusing. Is it possible to put dividing lines between the genes, so that it's clear how many genes are represented by each colored region?*

   We have completely redesigned figure 2 in a way that we believe vastly aids in data interpretation.

4. *For the RNASeq analysis shown in Figure 2b, did the authors also perform RNASeq analysis on the middle region of the worm, where chemoreceptor expression is not expected (or should be lower)? It would be nice to have this data as a control.*

   We unfortunately do not have these data. One issue is that the size of the head and tail tissues (~1 mm in length) are less than 5% of the entire length and contain very little RNA. Ideally, statistical comparisons to the more RNA-rich middle region (>20 mm) would require us sampling similar-sized tissue sections from the middle. We kept it simple in this first exploration, but are working towards an RNA tomography approach that will allow us to map genes across the entire body plan at higher resolution. We hope to present those data in a future publication.

5. *Figure 4: What is the scale for the chemotaxis assays? It looks like the worms would hardly need to move to get to the C or T regions. How motile are the worms? For the motility assay, can the authors provide data on the extent to which the worms disperse*

across the plate? They can clearly move out of the center region, but it's not clear how far they are capable of moving.

A scale has been added to Figures 4 and 7 to show distances. These distances were selected based on previous publications of filarial worm motility on agarose plates (see citations 22-25 in the manuscript). The worms are surprisingly motile on this surface and parasites can occasionally be found on the edges of the 6 cm plate within the 30 minute assay window. While they are capable of moving longer distances, they typically stay within 3 cm. These distances allow for robust and reproducible chemotaxis towards established cues, with almost all parasites finding their way to serum in every technical and biological replicate.

6. *Regarding Figure 5: a) Is the coiling response reversible, for example if the worm is put back at 37C? If it's not reversible, isn't it possible the response results from muscle or tissue damage, or some other more general response? b) Does adding NAM to L3s that have adapted to RT cause them to become more active by mimicking the effect of heat? c) I don't understand the description of sample sizes. Did the authors test only 3 worms per condition? Or does each biological and technical replicate involve multiple worms? If only 3 worms were used, this seems very low. Did the authors perform power analysis to get a sense of how many worms they should be testing? d) The coiling score seems like it could be made more rigorous. First, it's not explicitly stated how the coiling score is being determined. What are the criteria for rating worms on a scale of 0 to 5? The descriptions in the methods section is really vague. Also, the data would be more convincing if a more quantitative measure were used. If there are videos of the worms anyway, isn't it straightforward to measure curvature quantitatively?*

   The coiling response is indeed reversible; please see S4 Figure which shows 4/6 wells of parasite L3s exhibiting decreased motility after cooling and subsequent increased motility after warming. We have clarified the description of the replicates (lines 569-573). Each technical replicate is a well of 10-25 parasites, each biological replicate had a minimum of four technical replicates, and three biological replicates were performed. Furthermore, for the manual coiling analysis, three independent reviewers assigned a score for each technical replicate in all biological replicates. We have included as supporting info the document that was distributed to reviewers prior to scoring (S3 File). To support the qualitative analysis, we implemented a quantitative measure of motility using an optical flow algorithm that has been validated with these worms.

7. *Figure 8: I'm not sure what can be concluded from this experiment. Is there any evidence the channels are actually being expressed in C. elegans? It doesn't look like they were expressed using an SL2::GFP, making it difficult to determine whether the channels are non-functional in C. elegans or are not being expressed sufficiently. Given that it's hard to conclude much from this data, perhaps this figure can be moved to supplemental?*

To ensure that lack of rescue was not due to a lack of transgene expression, we performed qPCRs on the knock-out background strains and the transgenic strains (S10 Figure). We also greatly expanded this thread of experimentation to include new transgenes, strains, and sensory assays that substantially strengthens this section of the manuscript.

Minor issues:

1. *Starting off with a diagram of the life cycle of Brugia would be helpful for the non-expert.*

   A simple Brugia life cycle diagram has been added as an inset to Figure 1.

2. *Bottom of p. 9: Should this say, "These pathways have likely evolved to reflect the diversity of nematode life-history traits and environmental cues encountered by different *nematode* species" (i.e., "nematode" instead of "parasite")?*

   Thank you, this correction has been made (line 80-81).

3. *The expression data in Figure 2a is difficult to interpret. Is there a legend for the purple shading? Also, it's hard to see the purple shading when it's inside the circle, and it's confusing the way Figure 2b is discussed before this part of Figure 2a in the main text. It might be nice to separate out the life-stage expression data into a separate figure.*

   We have completely redesigned figure 2 in a way that we believe vastly aids in data interpretation. This new design also removes (A) and (B) and makes it better comport with the interpretation provided in the manuscript body.

4. *Is there expression data for the signaling proteins (tax-2, tax-4, osm-9, etc.) showing whether they are expressed in the head region (like what is shown in Figure 2b for the chemoreceptors)? This would provide some additional information about which of these genes is likely to have a chemosensory function in filarial nematodes.*

   In Figure 2 we are giving particular focus to *B. malayi* chemoreceptors, but your suggestion to incorporate TRPs and CNGs into the head/tail RNA-seq analysis is a good one, and it led us to an interesting observation regarding the potential for sex-specific interaction of TRPV subunits. We have added relevant transcript abundance values to the text in the results sections that describe the TRP and CNG phylogenies (lines 181-201).

5. *For Figure 3c-f, it would be helpful to indicate at the top of each figure which gene is shown.*

This is a helpful suggestion to ease reading and has been implemented.

6. *Figure 4: How many worms were typically used for each chemotaxis assay? Also, in Figure 4b, why is the mean given only for the untreated condition and not the NAM condition? For Figure 4e, are there untreated controls with freshly extracted parasites that were performed in parallel? It would be nice to have data for freshly extracted parasites and 1 DPE parasites that were tested in parallel shown side-by-side.*

   The number of worms used in each chemotaxis assay is provided in the methods section (8-10 L3s per plate). We have now added means for both conditions (NAM- and NAM+) in the plot. We did not perform the experiments for Figure 4E side-by-side with freshly extracted worms. Infections of jirds at the FR3, extractions of microfilaria, and infections of mosquitoes in our own insectary are precisely coordinated, so producing 1 DPE L3s on the same day as freshly extracted 14 DPI L3s would require reorganization of infection schedules at both locations. We were originally alerted to loss of chemotaxis phenotypes in day-old L3 larvae after we were unable to recapitulate published results (serum attraction). After switching to freshly-extracted L3s, we have recorded robust/reproducible responses to cues across all technical and biological replicates. We then went back and tested day-old L3 larvae (the data we have presented) to confirm that the difference was due to the length of time we waited post-mosquito extraction. While we didn't time the serum chemoattraction assays of fresh and day-old L3s on the same day, these assays were carried out in the same weeks, using the same plates, and using a common stock of serum.

7. *Figure 6a: Is there a reason not to label every point along the x-axis?*

   Not a good one! We've updated the x-axis labelling of figure 6A.

8. *Throughout the manuscript, it's not clear exactly how many worms were tested for each assay. Also, are the biological replicates different groups of mosquitoes and the technical replicates different trials? Were the different trials performed on the same day or separate days? How many mosquitoes were used for each biological replicate? Are the data points shown the means from each biological replicate?*

   We have added text to the methods sections and figure captions to clear up questions regarding replication and the number of worms/mosquitoes included in each trial (lines 549-554 and 569-573). For chemotaxis assays, 8-10 parasites were used per plate, and at least 3 plates were used per treatment group. Chemotaxis assays were repeated three times on different days (two times for 3-methyl-1-butanol) using parasites extracted from separate mosquito infections from different batches of mf. Each data point is the CI of a single plate, and means with error bars were calculated using data from all plates across all replicates. For coiling assays, 10-25 parasites were used per well, and at least 4 wells were used per NAM concentration. Coiling assays were repeated three times on different days using parasites extracted from separate mosquito infections from different

batches of mf. In this case, individual data points are not shown due to the large number per NAM concentration, but means and error bars were calculated using data from all wells across all replicates, with three manual scores for each well.
