# Supplementary material for: Genetic and functional diversification of chemosensory pathway receptors in mosquito-borne filarial nematodes
Source: PLoS Biol. 2020 Jun 8;18(6):e3000723. doi: 10.1371/journal.pbio.3000723 (PMC7302863; doi:10.1371/journal.pbio.3000723)
Supplement: Attachment — Submitted filename: Reviewer_Responses.pdf [file pbio.3000723.s023.pdf]

Dear Dr Zamanian,

Thank you very much for submitting a revised version of your manuscript "Genetic and functional diversification of chemosensory pathway receptors in mosquito-borne filarial nematodes" for consideration as a Research Article at PLOS Biology. This revised version of your manuscript has been evaluated by the PLOS Biology editors and by the original Academic Editor and reviewers 2, 3, and 4.

In light of the reviews (below), we are positive about your manuscript and are thus pleased to offer you the opportunity to address the remaining points from the reviewers in a revised version that we anticipate should not take you very long. We will then assess your revised manuscript and your response to the reviewers' comments and we may consult the reviewers again.

We expect to receive your revised manuscript within 1 month.

Reviewer 2

The authors have responded constructively and positively to my concerns. I think this is an important contribution to the literature and recommend that it now be processed for publication.

Reviewer 3

Review of manuscript #PBIOLOGY-19-02073R2 by Wheeler, Zamanian, et al. entitled "Genetic and functional diversification of chemosensory pathway receptors in mosquito-borne filarial nematodes"

This second revision is significantly improved and remains a thorough phylogenetic study of chemoreceptors and downstream signaling elements in Brugia malayi. Considering the need for novel interventions in human filariasis, their finding of a reduced and highly diverged repertoire of chemoreceptors compared to free-living nematodes represents a significant contribution to the literature on lymphatic filariasis and is consistent with the life history of this lymphatic dwelling filaria that inhabits either a mammalian or a mosquito host with only a brief transitional exposure to the external environment during the transition between emergence from the labial sheath of the mouthparts and invasion of the mosquito bite wound. Notably, the authors have discovered changes in expression patterns of B. malayi chemoreceptors that coincide with key migratory events and transitions between host and vector body compartments. Intriguingly, exposure to nicotinamide, an agonist of the sensory neuronal TRP channel OSM-9, suppresses outward migration of microfilariae from the midgut, suggesting that OSM-9-dependent chemotaxis is required for this vital migratory transition by the parasite. This is a testable hypothesis that could form the basis for future studies. Significantly, they also show that worms exposed to dsRNAs with sequence homology to the TRP channel encoding gene Bm-osm-9 and the CNG channel encoding gene Bm-tax-4 are impaired in their ability to execute diacetyl- and isoamyl alcohol-stimulated chemotaxis.

In this second revision, I believe that the authors have satisfactorily addressed most of the substantive issues that I noted in my review. In reading the rejoinder to other reviewers' comments it appears that they have similarly addressed substantive issues to the extent that this very challenging filarial system allows. I have only one substantive and one minor editing point to list with regard to this second revision.

Major Issues:

1. *The issue I raised about confirming efficient knock-down of target transcripts remains, as the authors were unable to collect sufficient parasite material either in the context of their experiments involving Bm-osm-9 and Bm-tax-4-specific knockdown or in the context of additional supporting experiments to accurately confirm suppression of target transcripts. I can accept that this technical hurdle is insurmountable at this time but urge the authors to consider devising approaches to addressing it in future projects and publications.*

   Thank you for your comments and understanding the technical difficulties of the *in vivo* RNAi protocol for filarial nematode larvae. We are working to further streamline the RNAi pipeline to decrease the amount of mosquito death after injection, increase the L3 prevalence in lab-infected mosquitoes, and thus increase the amount of parasite material/RNA available for experimentation and qPCR validation of transcript knockdown. We are also considering low-pass RNA-seq as a potential means of measuring KD and potential off-target effects.

Minor Issues:

1. *Strictly speaking the word "data" is plural, so issues with subject-verb agreement arising from its use as a singular are commonplace. The authors carefully addressed points in the main text relating to this. However, a few lapses are still seen in the figure captions in the second revision. Captions to Figures 4, 6, 7 and 8 all contain some variation on "Data….represents". It should be "Data…..represent". It might be a good idea to check any captions to supplemental figures along this line.*

   Thank you for catching these errors. These have now been corrected.

Reviewer 4

Overall, the authors have done a thorough job in addressing the reviewers' comments and the revised manuscript is much improved. I expect this paper will soon be a landmark in the field. While it would have been nice to see RNAi for another endogenous gene as a control to increase confidence in the RNAi data, I understand that these experiments are not feasible at this time. I have only a few remaining comments which I think should be addressed prior to publication.

Major Issues:

1. *In my opinion, the biggest issue remaining with the revised paper is with the rescue data shown in Figures 8 and 9.*

   First, I would suggest removing some of the inconclusive rescue data from the paper. Since using the unc-54 3' UTR didn't work, I don't think anything useful is gained by including this data. Also, for the diacetyl experiment, you still can't conclude anything since the C. elegans rescue didn't work. I don't think having this data in the paper is informative, and if it is included, I might at least move it to a supplemental figure.

   We agree that the data showing lack of rescue (i.e. with the *unc-54* 3' UTR or for the diacetyl chemotaxis) is less interesting as negative data and less relevant now that we have data from alternative constructs. We have retained two sentences (lines 325-8) about the initial use of the *unc-54* 3' UTR and moved these experiments showing a lack of rescue to a supporting figure (S11 Figure).

   Second, it looks like the B. malayi cDNA was used directly for the rescue experiment. However, expression of cDNA from other nematodes in C. elegans often requires the placement of internal syntrons in the cDNA. A syntron before the gene is sufficient for C. elegans cDNA expression, but often not for the expression of cDNA from distantly related nematodes. I would strongly suggest repeating this experiment using synthesized cDNA with internal introns, or at least using this approach going forward. From the data shown, I don't think anything can be concluded other than that partial rescue is possible in some cases.

   In our experience with several other receptor genes from *B. malayi*, we haven't had problems expressing in a range of tissues (sensory neurons, pharynx, body wall muscle) without internal introns. We have had constructs synthesized in the past with three internal introns, but never used them as the cloned parasite ORFs ended up rescuing in those contexts. We agree that it is possible in this case that the lack of *osm-9* rescue in AWA is driven by missing introns - we have therefore moved that data to a supporting figure as suggested (S12 Figure).

   Third, if the C. elegans osm-9 promoter fragment being used is the issue with the diacetyl experiment, there are AWA-specific promoters that could be used instead. If it's not possible to repeat these experiments at this point - I do realize these experiments are extremely difficult and time-consuming - I would at least be very careful about concluding anything beyond the fact that partial rescue occurred. In particular, I would remove the sentence about how partial rescue may suggest subfunctionalization, or at least qualify that this could be due to issues with transgene expression.

We agree with the reviewer that it would be ideal to iterate through more promoters and explore some of these possibilities, but it would be difficult to repeat all of these experiments a third time with a third set of constructs in the near future. However, we are planning experiments in the long term to more systematically explore some of the common issues of expressing clade III parasite genes in *C. elegans*. We also plan to investigate sensory receptor subunit interactions with the additional knowledge we gain. As for the neuron-specific promoters, when we first designed these experiments we initially planned on using *sra-6p* to express in the ASH neuron, but we aborted this line of experimentation when we found the partial rescue with the *osm-9* 3' UTR. In our view, the data provided is enough to support our primary conclusions, but we agree that suggesting the possibility of subfunctionalization is an over-interpretation and thus have removed this phrase as suggested (line 338).

2. *The methods section states that for the chemotaxis assays, assays were counted if C + T > 2. This seems very low. I doubt the CI is very meaningful when only two worms are involved in the calculation. I would suggest investigating this possible issue by repeating the calculations using only assays where C + T > 5 to see if the data look similar.*

    We changed the threshold such that at least 5 parasites move from the center and regenerated Figures 4 and 7. None of the conclusions changed with this more stringent threshold. We updated the methods (line 420), effect sizes (lines 215), and calculated means (lines 296-7) accordingly.

Minor Issues:

1. *In the legend for Figure 1, panels B and C appear to be switched.*
2. *Line 137: Change to, "that likely aid in copulation."*

    These have been corrected.

3. *Fig. 2: Can the authors speculate as to why there are so many chemoreceptors expressed in embryos?*

    It is possible that chemoreceptor transcripts buildup in embryos before release from the adult female. It is also very likely that there is low-level contamination of egg, embryo, microfilariae, and adult female RNA from crude surgical preparations. This is a known issue (Choi et. al., PLoS NTD 2011) and it is difficult for us to speculate given current limitations and lack of alternative approaches. The highly similar chemoreceptor expression pattern between embryos and adult females is suggestive of some contamination and we have added a sentence to the manuscript to reflect this caveat for this stage (lines 155-8). Tissue from other life cycle stages can be more easily and cleanly isolated.

4. *Figure 4E: axis label says "Indexi" instead of "Index."*
5. *Figure 5B: The text (lines 237-238) doesn't seem consistent with the figure and figure legend. The text makes it sound like the images in 5B show wild-type phenotypes at different temperatures, whereas the figure looks like it's showing control vs. NAM-treated.*

   These have been corrected.

6. *Figure 5: Why were one-sided t-tests used instead of two-sided t-tests?*

   We used one-sided tests in this case because we had the alternative hypothesis that NAM treatment of coiled worms would result in an increase in motility in comparison to non-treated worms. The use of two-sided tests would not alter the conclusions ($p < 0.05$ for all comparisons currently measured as significant, except $p = 0.067$ for 10 μM in the automated analysis).

7. *Figure 6B and F: Why were t-tests used instead of ANOVAs? It looks like 3 groups are being compared.*

   According to the reviewer's critique, we switched the test to use an ANOVA with Tukey's post hoc tests. This test slightly changes the conclusion of the blood-feeding experiment, showing that both 5 mM and 25 mM NAM cause a slight increase in the proportions of feeding mosquitoes (line 261-2). The conclusions drawn from Fig 6F do not change.

8. *If there's any way to get Figure 7 to fit in the normal orientation, that would be preferable.*
9. *Is there a reason not to use violin plots in Figure 7? I think the violin plots used elsewhere in the manuscript are a really nice way of representing the data.*

   The moving of some data to supporting info allowed us to convert all plots to violin plots in the normal orientation.

10. *Line 295-296: "To our knowledge, this is the first time that tax-4 or osm-8 have been shown to have a specific function in chemotaxis in a parasitic nematode." This is not quite correct. A recent paper showed a role for tax-4 in plant-parasitic nematodes using an RNAi approach:*

    Nagendrappa, S.K.T., Dutta, T.K., Chaudhary, S., von Reuss, S.H., Williamson, V., and Rao, U. (2019) Homologs of Caenorhabditis elegans chemosensory genes have roles in behaviour and chemotaxis in the root-knot nematode Meloidogyne incognita. Mol Plant Microbe Interact, e-pub ahead of print.

    Thank you for bringing this recent publication to our attention. This citation has been added (line 300).

11. Line 305 needs proof-reading.
12. Line 330: change “addition” to “additional”

These have been corrected.
