# Supplementary material for: Genetic and functional diversification of chemosensory pathway receptors in mosquito-borne filarial nematodes
Source: PLoS Biol. 2020 Jun 8;18(6):e3000723. doi: 10.1371/journal.pbio.3000723 (PMC7302863; doi:10.1371/journal.pbio.3000723)
Supplement: Attachment — Submitted filename: response.pdf [file pbio.3000723.s024.pdf]

We have made the changes enumerated below and have resaved our EPS files into Adobe Illustrator CC legacy format. Please let us know if there are outstanding issues.

Dear Dr Zamanian,

Thank you for resubmitting your manuscript "Genetic and functional diversification of chemosensory pathway receptors in mosquito-borne filarial nematodes" to PLOS Biology. We noticed a few remaining issues that will need to be addressed before your manuscript can be formally accepted. Your submission has been sent back to you to enable you to make these changes.

We would appreciate your prompt cooperation with our requests at your earliest convenience. If you have any questions or require clarification, please do not hesitate to get in touch.

MANUSCRIPT REQUIREMENTS

Please update your manuscript with the following changes:

1. Please ensure that all supporting information file names use the following format verbatim in the file inventory. The file names themselves must use an underscore rather than a space, i.e. S1_Fig.tif, S1_Table.xlsx, S1_Text.doc, S1_Data.xlsx, S1_PRISMA_Checklist.docx, etc. Each file type should be numbered starting at 1. Please also make sure that the file description matches the file name (e.g. S1_Fig.tif is listed as S1 Fig).

2. Please ensure that all supporting information legend titles use the following format verbatim in all in-text citations (including spelling and capitalization): S1 Fig, S1 Table, S1 Text, S1 Data, S1A Fig etc. Each file type should be numbered starting at 1.

FIGURE REQUIREMENTS

Please complete the following instructions regarding your figure files:

1. Please ensure that all main figures are referenced in the manuscript as Fig 1, Fig 2, etc. (including capitalization), rather than Figure 1, Figure 2, etc. Please note, however, that the file names themselves must not include the space, i.e. Fig1.tif, Fig2.eps.

Please use the following examples of naming conventions for citing multiple figures in your manuscript:

- Figs 3 and 4
- Figs 3, 4, and 7
- Figs 1-5
- Fig 3A and 3B
- Figs 3A and 7B

2. Please note that your figures do not currently comply with our publication requirements. For further instructions to assist with your Figure revisions, please refer to our Figure Guidelines at: http://journals.plos.org/plosbiology/s/figures.

If you need assistance, please contact us directly at graphics@jjeditorial.com so we can further help with this matter. Please send any relevant source files and tell us what application(s) and what operating system you are using.

We were unable to open Figures 1-9 and received the following error message: "Can't open illustration. The file was generated by a newer version of Illustrator and cannot be opened. Please resave it in a format compatible with your version of Illustrator." Please resave these files in a format compatible with Adobe Illustrator 2019.

You will find your submission in the 'Submissions Sent Back to Author' folder. Once you have made these changes, please re-approve the submission following the same steps as before.

Please do not hesitate to contact me should you have any questions.

Best wishes,

Jessica Hyde
Publications Assistant
PLOS Biology
